# CABE: A Cloud-Based Acoustic Beamforming Emulator for FPGA-Based Sound Source Localization

**DOI:** 10.3390/s19183906

**Published:** 2019-09-10

**Authors:** Laurent Segers, Jurgen Vandendriessche, Thibaut Vandervelden, Benjamin Johan Lapauw, Bruno da Silva, An Braeken, Abdellah Touhafi

**Affiliations:** 1 Department of Engineering Technology (INDI), Vrije Universiteit Brussel (VUB), 1050 Brussels, Belgium; jurgen.vandendriessche@vub.be (J.V.); thvdveld@vub.be (T.V.); benjamin.johan.lapauw@vub.be (B.J.L.); bruno.da.silva@vub.be (B.d.S.); an.braeken@vub.be (A.B.); abdellah.touhafi@vub.be (A.T.); 2 Department of Electronics and Informatics (ETRO), Vrije Universiteit Brussel (VUB), 1050 Brussels, Belgium

**Keywords:** cloud-based acoustic beamforming emulator, CABE, microphone array beamforming, FPGA microphone array beamforming emulator, delay-and-sum cloud-based emulator

## Abstract

Microphone arrays are gaining in popularity thanks to the availability of low-cost microphones. Applications including sonar, binaural hearing aid devices, acoustic indoor localization techniques and speech recognition are proposed by several research groups and companies. In most of the available implementations, the microphones utilized are assumed to offer an ideal response in a given frequency domain. Several toolboxes and software can be used to obtain a theoretical response of a microphone array with a given beamforming algorithm. However, a tool facilitating the design of a microphone array taking into account the non-ideal characteristics could not be found. Moreover, generating packages facilitating the implementation on Field Programmable Gate Arrays has, to our knowledge, not been carried out yet. Visualizing the responses in 2D and 3D also poses an engineering challenge. To alleviate these shortcomings, a scalable Cloud-based Acoustic Beamforming Emulator (CABE) is proposed. The non-ideal characteristics of microphones are considered during the computations and results are validated with acoustic data captured from microphones. It is also possible to generate hardware description language packages containing delay tables facilitating the implementation of Delay-and-Sum beamformers in embedded hardware. Truncation error analysis can also be carried out for fixed-point signal processing. The effects of disabling a given group of microphones within the microphone array can also be calculated. Results and packages can be visualized with a dedicated client application. Users can create and configure several parameters of an emulation, including sound source placement, the shape of the microphone array and the required signal processing flow. Depending on the user configuration, 2D and 3D graphs showing the beamforming results, waterfall diagrams and performance metrics can be generated by the client application. The emulations are also validated with captured data from existing microphone arrays.

## 1. Introduction

In recent years, advances in Micro ElectroMechanical Systems (MEMS) microphone technology and acoustic beamforming techniques allow for enhanced sound source localization in both acoustic and ultrasound frequency range [1,2,3]. Sound source localization based on microphone arrays have emerged and are used in various applications; ranging from ultrasound source localization [4], speech localization [5], binaural hearing aid for disabled people [6] and sonar [7]. Advances in embedded platform technologies allow the possibility to implement beamforming algorithms for microphone arrays on reconfigurable architectures such as Field Programmable Gate Arrays (FPGA) [3,8,9,10]. The placement of the microphones, the utilized algorithms and the amount of microphones determine the beamforming accuracy in terms of acoustic frequency resolution and spatial resolution. Estimating the response of a microphone array based on emulations prior to a hardware implementation has been achieved by da Silva et al. [8]. There the FPGA algorithms were emulated on a local computer in order to obtain optimized algorithms before the implementation on the hardware. Aside from calculating the directivity of the array in one direction, the 95% confidence interval of the directivity when steering in $360^{\circ }$ has been calculated. Matlab offers the possibility to calculate the response of a microphone array through the additional “Phased Array System Toolbox” [11]. Steckel et al. used ultrasound beamforming to develop and improve a 3D enabled sonar which allows to extract information of the environment using sparse arrays of microphones in conjunction with a single Polaroid emitter [4]. Sun et al. [12] proposes an improved Direction of Arrival (DoA) technique based on the generalized cross correlation algorithm in conjuction with a probabilistic neural network approach for enhanced sound source localization in noisy and reverberant rooms. Most beamforming tools allow to perform an analysis of a given microphone array along with the selected beamforming algorithm. In WaveCloud [13,14] an open source simulation tool for acoustic sound propagation in buildings is proposed. Users are free to download and install the tool. WaveCloud imports a 3D-stl file of the building and users can place sound sources in the simulation environment. All the previously discussed tools are executed locally and most are computational intensive and prohibits the use of the local machine for other tasks. Moreover, these tools are bound to a group of users having access to that particular machine.

In a recent movement, several companies offer cloud-based simulation alternatives to the well-established engineering tools. Simscale [15] offers an online simulation platform for Computational Fluid Dynamics (CFD) and Finite Element Analysis (FEA) used in structural engineering and thermal propagation in materials. This cloud-simulation tool is proposed in a free limited version as well as in paid versions for professional users. Waveller Cloud from KUAVA [16] is a simulation tool which allows to simulate acoustic propagation in prototypes such as the engines of cars, gearboxes, etc. They provide their tool as a Software as a Service (SAAS) and users pay a monthly fee or per CPU-hour cost. Aside of other cloud-based simulation tools, none of the tools provide the ability to simulate the beamforming of a user defined microphone array and to facilitate the implementation of FPGA by means of on the fly generated Very High Speed Integrated Circuit Hardware Description Language (VHDL) packages. In order to allow other researchers to grant access to the emulator without exposing the code and the associated learning curve, the previously proposed cloud-based platform where users can generate, modify and download emulations [17] is extended. The calculation of the beamforming and the generation of the VHDL packages are offered as a SAAS to facilitate local implementation onto FPGA boards.

This paper is structured as follows. In Section 2 we describe the background and state of the art of acoustic beamforming using arrays of microphones and extend the theoretical approach with non-ideal characteristics of the utilized microphones and required signal processing along with the mathematical principles. The beamforming quality of a microphone array based on the obtained results with a set of performance metrics are described in Section 3. In Section 4 the architectural overview of the cloud based emulator together with the implementation is described. In Section 5 the microphone arrays are detailed along with the obtained emulated output results which can be downloaded from the platform. The emulations are compared and validated with results obtained from acoustic captures in Section 6. In Section 7 the conclusions and future work implementations are proposed.

## 2. Acoustic Beamforming

Microphone arrays consist of several microphones placed in well defined patterns and come in different shapes and sizes offering different possibilities to locate a neighbouring sound source. The process to locate a sound source with a microphone array is referred as the beamforming method. Beamforming methods comprise several families of algorithms, including the Delay-and-Sum (D&S) beamformers [3,18,19], the Generalised Sidelobe Cancellation (GSC) beamformers [20,21,22], beamformers based on the MUltiple SIgnal Classification (MUSIC) algorithm [23,24] and beamformers based on the Estimation of Signal Parameters via Rotational Invariance Technique (ESPRIT) algorithm [25,26]. The most popular one—the Delay-and-Sum Beamformers—has been described by Dominguez et al. [3] by utilizing a microphone array consisting of 52 microphones. The D&S beamforming method can be applied in environments where uncorrelated noise is expected. This is especially the case when the microphone array is evaluated in an open field without any form of acoustic reverberations. The family of GSC beamformers also utilizes a D&S approach. The GSC method tries to alleviate the problem of correlated noise by estimating the amount of correlated noise in the environment. This estimation is later subtracted from the result of the regular D&S output. An adaptive variant is also proposed in [27]. The MUSIC and ESPRIT algorithms require more processing capabilities than the regular D&S but also offer a higher resolution in terms of Angle of Arrival (AoA) detection. Most of the algorithms described are presented with a more theoretical background, where the effects from the microphones can be considered ideal. Ideal microphones offer a one-to-one relationship between input and output without signal attenuation or amplification in certain frequency ranges. In some implementations the D&S beamforming algorithms are computed while taking into account the microphone effects [28,29]. Microphones—like any other mechanical device—do exhibit non-ideal characteristics regarding sampling, frequency response and introduced noise. Several types of microphones are available on the market, such as induction based microphones, condenser, piezocrystal, low-cost analog MEMS microphones and low-cost digital MEMS microphones. The manufacturing of a microphone array greatly depends on the type of microphones used. The processing requirements of signals coming from these microphones is also influenced by the selected microphone type. Therefore, the non-ideal characteristics of the microphones and the required signal processing during the evaluation of the microphone arrays are taken into account.

### 2.1. Delay and Sum Beamforming

The acoustic beamforming methods utilize acoustic signals from different microphones of the microphone array so that the AoA of a sound wave can be found. In order to steer in one particular direction, the algorithm will delay samples from the different microphones so acoustic signals bearing in the steering direction are amplified while acoustic signals coming from other orientations are suppressed. The principle of steering in direction $\vec{u}$ with the D&S beamforming can be expressed as [3]:

(1) $$o\left(\vec{u},t\right)=\sum_{m=0}^{M-1}s_{m}\left(t-\Delta_{m}\left(\vec{u}\right)\right)$$

Here, *M* corresponds to the number of microphones, $o(\vec{u},t)$ the resulting output signal in the unitary steering vector $\vec{u}$ and $s_{m}\left(t-\Delta_{m}\right)$ the samples from microphone *m* delayed by a time $\Delta_{m}$. Equation (1) expects a microphone array consisting of ideal microphones, i.e., microphones that transform mechanical waves into electrical signals without any form of distortion.

The time delay $\Delta_{m}$ for a microphone *m* in a given steering direction is obtained by computing the dot product between the vector ${\vec{r}}_{m}$ describing the location of microphone *m* in the array and the unitary steering vector $\vec{u}$. The delay factor is normalized by the speed of sound (*c*) in air.

(2) $$\Delta_{m}=\frac{{\vec{r}}_{m}\cdot \vec{u}}{c}$$

The delay and sum algorithm can be tranformed in the frequency domain as:

(3) $$O\left(\vec{u},\omega \right)=\sum_{m=0}^{M-1}S_{m}\left(\omega \right)\cdot e^{-j\omega \Delta_{m}\left(\vec{u}\right)}$$

### 2.2. Delay and Sum Beamforming with Non-Ideal Microphones

Equation (3) assumes ideal microphones delivering samples containing neither sampling noise nor frequency response effects. These non-idealities have to be taken into account when one desires to apply beamforming over a broad frequency range, i.e., also including the Helmholtz frequency regions [30] in case of ultrasound MEMS transducers. Therefore, Equation (3) is extended with a weight $W_{m}$ representing the frequency reponse of each microphone *m*.

(4) $$O\left(\vec{u},\omega \right)=\sum_{m=0}^{M-1}W_{m}\left(\omega \right)\cdot S_{m}\left(\omega \right)\cdot e^{-j\omega \Delta_{m}\left(\vec{u}\right)}$$

Digital Pulse Density Modulation (PDM) MEMS microphones oversample acoustic signals with a given rate and convert it into a 1-bit representation. Retrieving the original acoustic signal is done by filtering and decimating the PDM signal [31,32]. Many possible filter and decimation strategies have been utilized by da Silva et al. [9]. For simplicity, the filter and decimation stage are declared as an additional term dependant of ω, expressed as $H_{m}\left(\omega \right)$ for each microphone affecting the beamforming result. Equation (4) therefore results in:

(5) $$O\left(\vec{u},\omega \right)=\sum_{m=0}^{M-1}W_{m}\left(\omega \right)\cdot H_{m}\left(\omega \right)\cdot S_{m}\left(\omega \right)\cdot e^{-j\omega \Delta_{m}\left(\vec{u}\right)}$$

Dominguez et al. [3] described a method to compute the gain of a given microphone array in ideal circumstances. Their approach is extended with the non ideal-characteristics $H_{m}\left(\omega \right)$ and $W_{m}\left(\omega \right)$. The total gain of the microphone array in the desired frequency range can be computed by assuming that the array is exposed to a single monochromatic wave emitted from the far-field region. Such a wave can be approximated by the planar wave equation:

(6) $$\psi \left(\vec{v},t\right)=A\cdot cos\left(\vec{k}\cdot \vec{v}-\omega \cdot t+\phi \right)$$

where $\vec{k}$ represents the wave vector, $\vec{v}$ the vector representing the position of acoustic capturing, *A* the amplitude of the wave and ϕ the phase offset of the wave. In air, the wave vector is the same as the wave propagation direction ${\vec{u}}_{0}$ of the wave front while its magnitude is the wave number *k* so:

(7) $$\vec{k}=k\cdot {\vec{u}}_{0}=\frac{\omega }{c}\cdot {\vec{u}}_{0}$$

Equation (5) can be rewritten in function of the incidence direction of the planar wave $-{\vec{u}}_{0}$ and the current steering angle $\vec{u}$ so:

(8) $$O\left(\vec{u},\omega \right)=\sum_{m=0}^{M-1}W_{m}\left(\omega \right)\cdot H_{m}\left(\omega \right)\cdot S_{m}\left(\omega \right)\cdot e^{jk\cdot {\vec{r}}_{m}\left({\vec{u}}_{0}-\vec{u}\right)}$$

The array pattern $AP(k,{\vec{u}}_{0},\vec{u})$ [3] is identified by Equation (9) and delivers the theoretical beamforming pattern of the array.

(9) $$AP\left(k,{\vec{u}}_{0},\vec{u}\right)=\sum_{m=0}^{M-1}e^{jk\cdot {\vec{r}}_{m}\left({\vec{u}}_{0}-\vec{u}\right)}$$

One can define a frequency shaping function $F_{m}\left(\omega \right)$ for microphone arrays composed of non ideal microphones. This function consists of the response of the microphones $W_{m}\left(\omega \right)$ and the filter chain $H_{m}\left(\omega \right)$ applied during signal filtering at the beamforming stage. Depending on the application requirements, one can define $H_{m}\left(\omega \right)$ so the effects $W_{m}\left(\omega \right)$ are equalized over a broad frequency range. Simultaneously, $H_{m}\left(\omega \right)$ can also enable to filter out undesired frequencies in order to target a specific frequency range.

(10) $$F_{m}\left(\omega \right)=W_{m}\left(\omega \right)\cdot H_{m}\left(\omega \right)$$

### 2.3. Computing the Steered Response Power

Dominguez et al. [3] defined the output steered response power of the microphone array as a function of observed surrounding signals. The output signal $O(\vec{u},\omega )$ delivers the summed samples at steering orientation $\vec{u}$ for signals emitted around the microphone array. If a microphone array is exposed to a given broadband signal $S_{i}$ at steering vector $\vec{u}$, one can identify *N* different frequencies $\omega_{i}$ with amplitude $a_{i}$ of the signal resulting in an output signal $O(\vec{u},S_{i},\omega )$ so:

(11) $$O\left(\vec{u},S_{i},\omega \right)=\sum_{i=0}^{N-1}a_{i}\left(\vec{u}\right)\cdot S_{i}\left(\vec{u},\omega_{i}\right)$$

The microphone and filtering shaping functions $F_{m}\left(\omega \right)$ impact the output steered signal so that Equation (11) is rewritten as:

(12) $$O\left(\vec{u},S_{i},\omega \right)=\sum_{m=0}^{M-1}F_{m}\left(\omega \right)\sum_{i=0}^{N-1}a_{i}\left(\vec{u}\right)\cdot S_{i}\left(\vec{u},\omega_{i}\right)$$

By considering that the microphone array is placed in an open, reverberation free environment surrounded by *K* different broadband sound sources, one can compute the total acoustic signal observed by the microphone array as:

(13) $$O\left(\vec{u},S,\omega \right)=\sum_{m=0}^{M-1}F_{m}\left(\omega \right)\sum_{i=0}^{K-1}O\left(\vec{u},S_{i},\omega \right)$$

From here, the output power $P(\vec{u},S,\omega )$ of the microphone array in each steering direction $\vec{u}$ can be expressed as the sum of all squares of the computed steered output samples $O(\vec{u},S,\omega )$ in each steering direction $\vec{u}$.

(14) $$P\left(\vec{u},S,\omega \right)=\frac{1}{2\pi }\int_{-\infty }^{\infty }{\left|O(\vec{u},S,\omega )\right|}^{2}d\omega$$

### 2.4. Acoustic Beamforming on FPGA–Discrete Sampling

The above descriptions hold for acoustic signals considered in a continuous domain. However, each microphone captures acoustic signals at discrete time intervals and positions. The position of the microphone corresponds to the vector $\vec{r}$, while the time of capturing $t_{c}$ is represented by $t_{c}=n\cdot T$, where *T* is the period between 2 consecutive samples and *n* the *n*th sample of measuring. Therefore, the steering output of Equation (1) with delay $d_{n}$ is rewritten as:

(15) $$o\left(\vec{u},nT\right)=\sum_{m=0}^{M-1}s_{m}\left(\left(n-d_{n}\right)\cdot T\right)$$

so $o[\vec{u},n]$ becomes:

(16) $$o\left[\vec{u},n\right]=\sum_{m=0}^{M-1}s_{m}\left[n-\Delta_{m}^{\prime }\right]$$

Microphone manufacturing involves several processes which are performed within a given tolerance margin. Different microphones will exhibit different signal responses. One of these differences concerns the transduction time between acoustic capturing at the aperture hole and the transmission at the microphone’s output [33]. The spreading between the microphone with the shortest time delay (i.e., zero delay) and a given microphone in the array is given by a constant sample delay $\alpha_{m}$. This constant does not immediately influence the beamforming quality for lower frequencies (i.e., less then 5 kHz). However, applying D&S on signals with higher frequencies (i.e., ultrasound) may result in inaccurate or even false beams. Equation (16) is expanded so that:

(17) $$o\left[\vec{u},n\right]=\sum_{m=0}^{M-1}s_{m}\left[n-\Delta_{m}^{\prime }-\alpha_{m}\right]$$

The time delay $\Delta_{m}^{\prime }$ for a given microphone *m* can be obtained by computing the dot product between the position of the microphone ${\vec{r}}_{m}$ and the unitary steering vector $\vec{u}$ normalized to the speed of sound. Since the signal is sampled at discrete time intervals, Equation (2) is multiplied by the sampling frequency $f_{s}$ (or $\frac{1}{T_{s}})$ and the result is rounded to the nearest integer value. The obtained delay $\Delta_{m}^{\prime }$ corresponds to a sample index. We assume that $\alpha_{m}$ from the above equation is not frequency dependent and constant over a short amount of time. This sample delay value $\alpha_{m}$ can thus mostly be cancelled out by compensating it with a value $T_{delay,m}$ during the delaying process.

(18) $$\Delta_{m}^{\prime }=round\left(f_{s}\cdot \left(\frac{{\vec{r}}_{m}\cdot \vec{u}}{c}+T_{delay,m}\right)\right)=round\left(\frac{1}{T_{s}}\cdot \left(\frac{{\vec{r}}_{m}\cdot \vec{u}}{c}+T_{delay,m}\right)\right)$$

For sufficiently high sampling frequencies $f_{s}$ the above equation can be rewritten so that:

(19) $$\alpha_{m}\approx f_{s}\cdot T_{delay,m}$$

and

(20) $$\Delta_{m}^{\prime }\approx round\left(f_{s}\cdot \frac{{\vec{r}}_{m}\cdot \vec{u}}{c}\right)+\alpha_{m}=round\left(\frac{1}{T_{s}}\cdot \frac{{\vec{r}}_{m}\cdot \vec{u}}{c}\right)+\alpha_{m}$$

which results back in the original Equation (16). This approximation can be applied on PDM microphones due to the higher sampling rates.

In the remainder of the text we will assume that the delay $\alpha_{m}$ induced by each microphone can be compensated and omitted from the equations, unless otherwise specified.

Equation (15) can be transformed into the *z*-domain by applying following *z*-domain delay identity

(21) $$Z\left(f\left[n-d\right]\right)=F\left(z\right)\cdot z^{-d}$$

so that, along with the frequency shaping of the microphones and the filter during delay and sum:

(22) $$O\left(\vec{u},z\right)=\sum_{m=0}^{M-1}W_{m}\left(z\right)\cdot H\left(z\right)\cdot S_{m}\left(z\right)\cdot z^{-\Delta_{m}\left(\vec{u}\right)}$$

The complete processing will be computed on embedded systems such as FPGAs. These platforms typically compute signals with filters where a finite precision such as fixed-point or integer format is used. Also, the quantization step at the Sigma-Delta Modulation (SDM) stage introduces a given amount of noise at the final stage calculations [34,35,36].

The non-linear signal dependant error $\xi (\vec{u},z)$ can be described as the error induced by the complete chain of computations and conversion so that Equation (22) can be written as:

(23) $$O\left(\vec{u},z\right)=\sum_{m=0}^{M-1}W_{m}\left(z\right)\cdot H\left(z\right)\cdot S_{m}\left(z\right)\cdot z^{-\Delta_{m}\left(\vec{u}\right)}+\xi \left(\vec{u},z\right)$$

The error $\xi (\vec{u},z)$ defines the total error compared to calculations with an infinite precision. The error $\xi (\vec{u},z)$ can be estimated by computing the difference between the total beamforming response of a given array in infinite precision and in the desired fixed point precision intended for embedded computations. If the infinite precision computations is defined with $O{\left(\vec{u},z\right)}_{inf}$ and the fixed point computations with $O{\left(\vec{u},z\right)}_{fix}$, then $\xi (\vec{u},z)$ can be formulated as:

(24) $$\xi \left(\vec{u},z\right)=O_{inf}\left(\vec{u},z\right)-O_{fix}\left(\vec{u},z\right)$$

Equation (22) can be rewritten so that $O(\vec{u},z)$ is a function of the incidence angle ${\vec{u}}_{0}$ of a monochromatic wave and the steering angle $\vec{u}$.

(25) $$O\left(\vec{u},z\right)=\sum_{m=0}^{M-1}W_{m}\left(z\right)\cdot H_{m}\left(z\right)\cdot S_{m}\left(z\right)\cdot z^{\frac{1}{c}\cdot {\vec{r}}_{m}\cdot \left({\vec{u}}_{0}-\vec{u}\right)}+\xi \left(\vec{u},z\right)$$

Similarly to Equation (14), the average power $P(\vec{u},S,z)$ of the output in a given steering orientation $\vec{u}$ for a duration of *L* sample periods is expressed as:

(26) $$P\left(\vec{u},S,z\right)=\frac{1}{L}\sum_{i=0}^{L-1}{\left|O_{i}\left(\vec{u},S,z\right)\right|}^{2}$$

The intention to obtain beamforming on embedded platforms such as FPGAs implies the possibility to compute with a finite precision which introduces quantization noise. This can be expressed by adding a $\xi^{\prime }\left(\vec{u},S,z\right)$ component to Equation (26) so that:

(27) $$P\left(\vec{u},S,z\right)=\frac{1}{L}\sum_{i=0}^{L-1}{\left|O_{i}\left(\vec{u},S,z\right)\right|}^{2}+\xi^{\prime }\left(\vec{u},S,z\right)$$

In a similar manner, the error $\xi^{\prime }\left(\vec{u},S,z\right)$ can be estimated by comparing the resulting power output when using infinite precision $P_{inf}\left(\vec{u},S,z\right)$ with results obtained when using finite fixed point precision $P_{fix}\left(\vec{u},S,z\right)$ so that $\xi^{\prime }\left(\vec{u},S,z\right)$ can be expressed as:

(28) $$\xi^{\prime }\left(\vec{u},S,z\right)=P_{inf}\left(\vec{u},S,z\right)-P_{fix}\left(\vec{u},S,z\right)$$

## 3. Performance Metrics

Depending on the intended applications, microphone arrays come in several shapes and sizes with a varying number of microphones. The placement of the microphones greatly affects the response of the array and thus the effectiveness of the main lobe. The main lobe generally represents the DoA of the acoustic wave to the microphone array. However, aside of the main lobe, many side and grating lobes of different amplitudes can occur. Researchers have tried to minimize the effects of the undesired lobes and therefore developed several metrics describing how well an array performs to a given acoustic source. These metrics can be seen as a complementary set qualifying the possibility to detect the AoA of a given sound source. Therefore, the proposed metrics are described and extended.

### 3.1. Beamwidth (BW)

A first metric described by Kelly et al. [37] is the BeamWidth (BW) of the main lobe. The beamwidth of the main lobe is strongly related to the acoustic frequency and decreases with increasing frequency. The beamwidth is defined as the region enclosed by the points of local minima around the maximum of the beam where the minima are at least −12 dB lower then the maximum of the beam (i.e., $\frac{P_{max}\left(\vec{u},S,z\right)}{4}$). The beamwidth is generally computed for a 2-dimensional response in $\theta \in [0,360^{\circ }]$. Here, this principle is extended to the ratio between the area $A_{BW}\left(\Omega \right)$ of the −12 dB values between α and β of the main beam and the total region of interest A(Ω) between *a* and *b* (Equation (29)),

(29) $$BW=\frac{\int_{\alpha }^{\beta }A_{BW}\left(\Omega \right)d\Omega }{\int_{a}^{b}A\left(\Omega \right)d\Omega }$$

where Ω represents the spherical coordinates of the azimuth θ and the elevation ϕ. This region of interest comprises all steering angles used for the result, and is bounded by $\theta \in [\theta_{a},\theta_{b}]$ and $\phi \in [\phi_{a},\phi_{b}]$ aperture. A smaller beamwidth generally corresponds to a higher probability of localizing a sound source at a given AoA. This is especially the case when the beamwidth is computed for the main lobe.

### 3.2. Peak Side Lobe Level (PSLL)

The Peak Side Lobe Level (PSLL) is another metric used by Sun et al. [38] and Caorsi et al. [39] and corresponds to the ratio between the main lobe $P_{m}\left(\vec{u},S,z\right)$ highest power value and the highest second peak power value $P_{sl}\left(\vec{u},S,z\right)$. This metric is generally given in a dB scale and is stated as follows:

(30) $$PSLL=20\cdot log_{10}\left(\frac{P_{sl}\left(\vec{u},S,z\right)}{P_{m}\left(\vec{u},S,z\right)}\right)$$

Lower PSLL values (PSLL∈[0,−∞)) correspond to a higher probability of finding a sound source in a given direction. A positive PSLL value however indicates that the current steering vector does not correspond to the main lobe detected by the beamforming.

### 3.3. Integrated Side Lobe Level (ISLL)

The Integrated Side Lobe Level (ISLL) is another metric proposed by Kelly et al. [37] and allows to compute the ratio between the area outside the main lobe between α and β and the area of the response of the array over the complete area of interest for a 2D response. Here, this principle is extended to 3D by:

(31) $$ISLL=\frac{\int_{\alpha }^{\beta }P_{\alpha ,\beta }\left(\vec{u},S,z\right)d\Omega }{\int_{a}^{b}P_{a,b}\left(\vec{u},S,z\right)d\Omega }\times 100\%$$

Here, the total region of interest is between *a* and *b* and Ω is a function of the azimuth angle θ and the elevation angle ϕ. Boundaries α and β can be chosen arbitrarily. Often, the −3 dB beamwidth boundaries are taken. A lower value for the ISLL combined with a narrow beam area between α and β is desired.

### 3.4. Focal Index (FI)

The aforementioned metrics sometimes lack of representing the quality of a given beam pattern efficiently. E.g., it is possible that the PSLL returns a 0 dB value, while the main lobe and the second lobe share the same power value, but with optimal beam pattern in other steering orientations. Kelly et al. [37] try to alleviate the shortcomings of these metrics by proposing the Focal Index (FI).

This metric is adapted here to take the steered response power value into account and is given by:

(32) $$FI=\left(\frac{A_{\alpha ,\beta }}{A_{a,b}}\right)\left(1-\left|\sqrt{P_{\left(1-\alpha ,\beta \right)}^{\prime }\left(\vec{u},S,z\right)}\right|\right)\left(1-PSLL_{lin}\right)$$

The ratio between $A_{\alpha ,\beta }$ and $A_{a,b}$ defines the ratio between respectively the acceptance area of the beam and the total area of interest. $PSLL_{lin}$ is the linear representation of the PSLL defined in Equation (30). The factor $\left|\sqrt{P_{\left(1-\alpha ,\beta \right)}^{\prime }\left(\vec{u},S,z\right)}\right|$ corresponds to the average normalized beam pattern of the array outside the acceptance area. A higher value of the FI∈[0,1] represents a higher probability of finding a sound source in the direction defined by the area between α and β. However, a negative FI at the current expected location of a sound source corresponds to another lobe being detected as more important. This also corresponds to a positive PSLL value.

### 3.5. Directivity Index (DI)

The last metric discussed here is the Directivity Index (DI) which describes the ratio between the power pattern in a given steering angle and the average power pattern in all steering angles and is adapted from [3,18] as

(33) $$DI=\frac{P({\vec{u}}_{0},S,\omega )}{\frac{1}{A_{a,b}}\int_{a}^{b}P\left(\vec{u},S,z\right)d\Omega }$$

where $A_{a,b}$ denotes the area of interest between *a* and *b* and $P({\vec{u}}_{0},S,z)$ the power obtained from the steering direction from which the sound source is susceptible to emit. A higher DI corresponds to a higher probality of finding an acoustic sound source in that particular steering angle.

### 3.6. Steered Metrics

The above mentioned metrics give an estimation of the performance of a given microphone array when steering at a particular orientation to a particular emitting sound source. Sound sources may not be located in the steering orientation of choice but in any random orientation. In order to evaluate a given microphone array to a given sound source emitting at any point in space, all the metrics are computed on the closest steering angle to the direction of arrival of a discrete sound source, while the sound source is moved over several discrete positions around the microphone array. The average metric, the 75% (i.e., Q3 factor) interval and the minimum and maximum observed metric are computed. The Q3 factor is obtained by computing the Median Absolute Deviation (MAD) on the obtained values, with $\bar{X}$ the expected average of the metric and $X_{\vec{u}}$ the value on steering angle $\vec{u}$ of the metric.

(34) $$MAD=median\left(\left|X_{\vec{u}}-\bar{X}\right|\right)$$

## 4. CABE: Cloud-Based Acoustic Beamforming Emulator

### 4.1. Architectural Overview

To facilitate beamforming computations in the research group, the CABE platform is developed which consists of 3 major parts. In the back-end several multi-core computers perform the beamforming computations (Emulator). The second part consists of a webserver running the User Web Interface and the Emulator Task Manager ensuring the connection between the back-end and the users. In the last part, users utilize the CABE client application to initiate new beamforming computations and to visualize the results.

**Figure 1:**
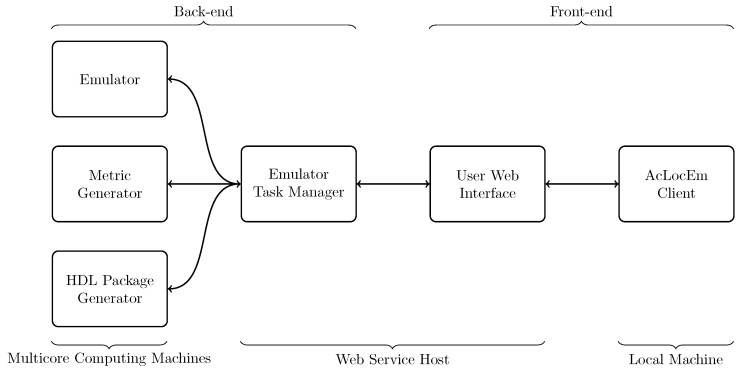
Architectural overview of CABE. The applications on the left side reside on several computing machines which compute results of beamforming, metrics and generate FPGA Hardware Description Language (HDL) packages. The Task Manager and the User Web Interface allow to queue and schedule new tasks while the CABE client allows to generate new emulation tasks and to visualise results. The back-end applications are not visible towards users.

The computers running the emulator also offer the possibility to compute the performance metrics for an emulated microphone array through the Metric Generator. The emulator computers also have an Hardware Description Language (HDL) package generator to generate HDL packages for implementations on FPGA. The User Web Interface facilitates the connection between the client application and computing machines in the back-end. This interface also schedules the requests of the users via the Emulator Task Manager. The communication between the entities is performed by means of RESTful Application Programming Interface (API) calls including the transfer of files. A general overview of the system is presented in Figure 1. All back-end parts and client applications are developed in C++ using standard libraries. The Web User Interface and Task Manager are developed in PHP as a web service so that access for both client and back-end applications are facilitated. Calculating beamforming algorithms can be compute-intensive and the processing time depends on the number of microphones, the number of steering angles and the number of sound sources. Therefore, the platform takes advantage of utilizing several concurrent back-end multicore computing machines. All back-end applications and web services are running on Ubuntu 18.04 LTS servers while the client application can be used on Linux and Windows machines. The cloud-based platform can be found at https://projects.rapptor.vub.ac.be/CABE/.

### 4.2. Emulator

**Figure 2:**
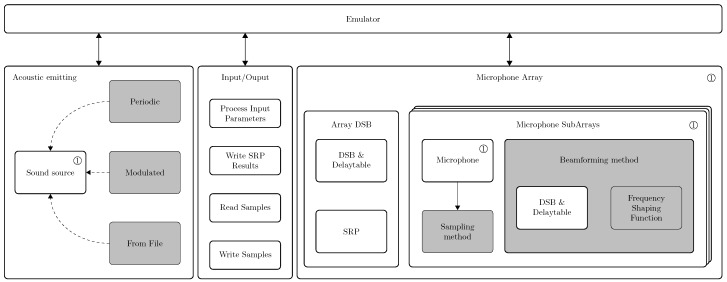
Overview of the emulator application which computes the beamforming on a given microphone array. The emulator is composed of 3 major parts. The first part ensures proper acoustic information generation, the second part computes the beamforming while the last part handles file operations. Modules marked with ‘1’ contain time and position information. Grayed out modules denote modules to be inherited by shared objects, allowing a more flexible implementation of features. A microphone array can also define multiple subarrays.

The emulator computes the responses of a user defined microphone array along with a set of processing constraints and a set of defined acoustic sources. The emulator is designed following a modular architecture so that currently researched algorithms can be included, but also allowing easy integration of new methods in the future. The general overview of the emulator is given in Figure 2. Shared objects are used so that the source code can be subdivided into smaller manageable libraries. This usage is intended for the sound sources, the beamforming methods and the frequency shaping methods since virtually any combination of these are possible. The general processing flow of the emulator is categorized in 5 major sequential steps:

1. processing input parameters,
2. microphone array response computation,
3. generating output response files,
4. computing metrics,
5. generating HDL package.

Steps 4 and 5 are optional and are delivered at the end of the processing flow. These steps can also directly be requested in case Steps 1 to 3 have already been carried out. In the next paragraphs, the processing flow of the emulator up to obtaining the steered response power, the performance metrics and the HDL package generation are detailed.

### 4.3. Processing Input Parameters (Step 1)

The emulator is capable of computing the beamforming response for a user defined microphone array with a custom set of parameters. These parameters and the layout of the microphone array in the three dimensional space are stored into configuration files including a simulation file, a microphone array file with the microphone types and the definitions of the subarrays for power savings [10]. There is also a file for the appropriate Digital Signal Processing (DSP) beamforming algorithms together with the chosen steering pattern. Optionally a file containing sound sources can also be provided. All configuration files are defined using the YAML Ain’t Markup Language (YAML) format; a human readable data serialisation format [40] and are linked in a main simulation file.

### 4.4. Microphone Array Response Computation (Step 2)

In Section 2 we described the mathematical background of microphone arrays. In this paragraph these principles are extended to embedded systems computing beamforming methods which sample acoustic signals in the discrete time domain. Moreover, since the development and the implementation of these algorithms targets FPGAs, the errors induced by signal quantization and errors related to computation with finite precision need to be estimated.

#### 4.4.1. Acoustic Capturing

Each acoustic source *i* with position ${\vec{P}}_{i}$ emits a given acoustic wave starting from a time $t_{0}$. Each of the microphones in the array captures acoustic waves at position ${\vec{r}}_{m}$ and sample time $t_{s}=\frac{n}{f_{s}}=n\cdot T$. By calculating the time of flight between each acoustic source and each microphone, one can obtain the total received signal $S_{m}\left[n\right]$ via superposition (Equation (35)).

(35) $$S_{m}\left[n\right]=\sum_{i=0}^{N-1}Source_{i}\left[\frac{n}{f_{s}}-\frac{\left|{\vec{r}}_{m}-{\vec{P}}_{i}\right|}{c}-T_{delay,m}\right]$$

Here, the condition $\frac{n}{f_{s}}-\frac{\left|{\vec{r}}_{m}-{\vec{P}}_{i}\right|}{c}-T_{delay,m}\geq t_{0}$ must be met (i.e., causal systems) so that an acoustic wave emitted from a sound source is captured. The sample obtained can be considered ideal and needs to be shaped according to the specifications of the microphones. Therefore, the microphone characteristics are taken into consideration during the emulation. This is done in two consecutive steps:

1. In the first step a frequency response shaping on the acoustic signal is applied by means of a convolution filter. This shaping is performed in time domain for easier streaming of samples. The frequency response of each microphone type is converted into FIR coefficients by the frequency sampling method [41].
2. The second step consists of converting the output of the frequency shaping function into the right output format proposed by the microphone. Currently supported formats include double precision to mimick analog samples, Pulse Coded Modulation (PCM) and Pulse Density Modulation (PDM). Conversion to PDM format needs to be carefully chosen to match the microphone’s characteristics. Here 4th and 5th order Sigma-Delta Modulation (SDM) converters with an Over Sampling Ratio (OSR) between 15 and 64 are generally used [31,42,43]. The appropriate architectures are designed with the Delsig Matlab toolbox [44].

In case the signal amplitude overloads the threshold of the microphone’s sensitivity, the acoustic signal could produce uncorrelated results to the original signal. Therefore, signals with amplitude values beyond the sensitivity level are clipped to the sensitivity level [45].

#### 4.4.2. Delay-and-Sum

The delay and sum principle is based on the assumption to delay a discrete acoustic information based on sample indices. All necessary delaying values for each steering orientation are generated beforehand and stored in a delay table. Each row corresponds to a given steering orientation while each column corresponds to a given microphone of the (sub)array. Each cell of the table thus corresponds to the delay per steering angle per microphone. The relationship for each delay in Equation (18) is adapted to avoid negative indices causing non causal signals. Therefore, for each steering orientation $\vec{u}$ the delay table $D[\vec{u},m]$ is reorganized so that:

(36) $$D\left[\vec{u},m\right]=D\left[\vec{u},m\right]-min\left[D[\vec{u},:]\right]$$

where $min\left[D[\vec{u},:]\right]$ and *m* respectively denote the minimal value of each row and microphone *m*.

**Figure 3:**
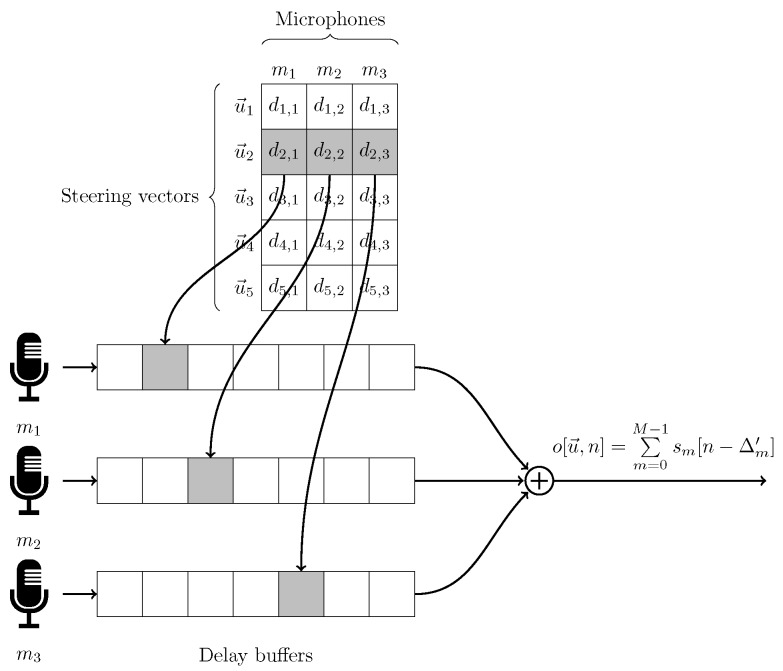
Delay and sum algorithm using a delay table. Grayed out cells of the delay buffers are summed together to form a new sample at the output.

An indexable ring buffer with a length equal to the maximum delay value is utilized. A sum of all the delayed samples per steering orientation is given per sample interval. Figure 3 depicts the processing flow of the delay and sum algorithm, along with the delay table.

#### 4.4.3. Steering Vectors

**Figure 4:**
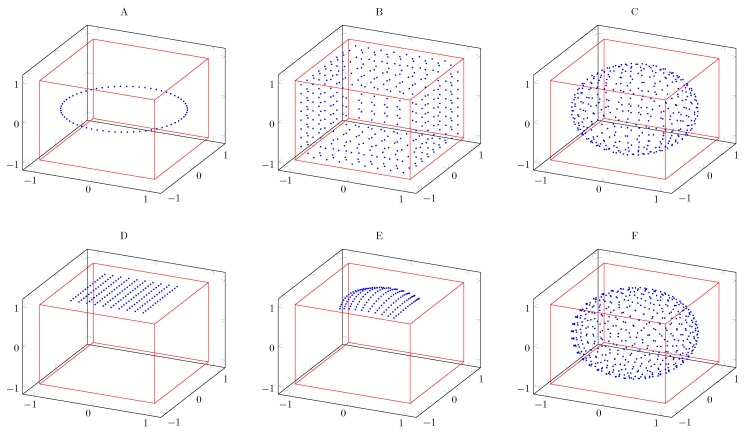
Vectors distributed following the (**A**) ‘Equalpolar Distribution’, (**B**) ‘Hypercube Distribution’, (**C**) ‘Hypercube Distribution Normalized’, (**D**) ‘Hyperplane Distribution’, (**E**) ‘Hyperplane Distribution Normalized’ and (**F**) ‘Fibonacci Lattice’.

The delay and sum algorithm relies on the steering vectors $\vec{u}$ to perform beamforming in different orientations. Four families of steering orientations are implemented (Figure 4):

- Equalpolar Distribution (A): 2D steering vectors in an equal radial pattern on one of the Cartesian planes. A start and stop angle can be provided limiting the “view area” of the microphone array.
- Hypercube Distribution (B and C): 3D steering following a hypercube distrubution. The distribution can be on the cube itself or can be normalized to a unit sphere.
- Hyperplane Distribution (D and E): enables to steer following a grid pattern onto one of the planes of the hypercube method. A normalized pattern can also be used.
- Fibonacci Lattice (F): 3D steering following the Fibonacci lattice distribution [46]. Here only the spherical distribution is available.

#### 4.4.4. Delay-and-Sum between Subarrays

Each microphone array can be composed of several groups of microphones forming subarrays. One can compute the proper delay table for each microphone subarray such as provided by Equation (36). When combining the output signals $O(\vec{u},z)$ however, all subarrays need to be aligned properly to avoid signal degradation. Therefore, a delay table containing the delays per steering vector $\vec{u}$ and per subarray is computed via a two-steps procedure.

- For each subarray *s*, the minimum dot product $D[\vec{u},s]$ (Equation (37)) between the steering vector $\vec{u}$ and the positions ${\vec{r}}_{m}$ of all microphones is computed. The minimal dot product is indexed in a temporary table. This step is repeated for all steering orientations.
- At the level of the main array, the delay table is computed by applying Equation (38) on the obtained distances from the subarrays for each of the steering orientations.
  (37) $$D\left[\vec{u},s\right]=min\left[round\left(f_{s}\cdot \left(\frac{\vec{u}\cdot {\vec{r}}_{m}}{c}+T_{delay,m}\right)\right)\right]$$
  (38) $$D\left[\vec{u},s\right]=D\left[\vec{u},s\right]-min\left[D[\vec{u},:]\right]$$

#### 4.4.5. Signal Demodulation and Frequency Shaping Function

The last stage of the computations includes the frequency shaping functions which allows to choose the desired frequencies at the end of the computations. Several strategies have been proposed in the literature for the various types of microphones. Depending on the type of microphone, decimating the signals before equalization has to be performed.

##### Analog MEMS and Condenser Microphones

Analog microphones are sampled with Analog to Digital Converters (ADCs) and streamed in PCM format in the D&S algorithms [47]. One strategy consists in filtering the samples with a low-pass Finite Impulse Response (FIR) filter before displaying the result [48]. Netti et al. however [49] propose an adaptive FIR filter technique in conjunction with a D&S to detect the position of vehicles towards environmental noise monitoring. Zimmermann et al. [50] presented a real-time approach by decimating the samples with a Cascaded Integrator Comb (CIC) filter followed by a FIR filtering for an acoustic camera.

##### Digital PDM Microphones

PDM (MEMS) microphones typically oversample the signals with a factor of 15 up to 64. Acoustic decimation and filtering must be applied to retrieve the acoustic information. Several strategies have been carried out [47]. A common strategy consists in decimating the PDM signal by means of a CIC filter. CIC filters offer the advantage of avoiding the required number of multiplications of a FIR filter by only adding and subtracting the samples during filtering. This filter is also commonly known as the “sync” filter due to its resulting frequency response [51]. Therefore, a lower order CIC filter is used in conjunction with another family of filters. Some proposed methods include further decimation by a cascade of halfband filters [52,53]. Halfband filters are a specialized subset of FIR filters where half of the coefficients are zero and allow to decimate the signals by removing signal components beyond a quarter of the original sampling frequency (i.e., after CIC filtering) in order to satisfy the Nyquist theorem. A lower order FIR compensation filter equalizes the distortion induced by the CIC filter. Another strategy consists by immediately filtering the output of the CIC filter by a FIR compensation low-pass filter [54].

##### Signal Demodulation and Signal Shaping in the Emulator

A few strategies are provided in the current emulator, including the filtering of PCM samples with a FIR filter and the demodulation of PDM samples. Strategies including a CIC filter followed by a cascade of halfband filters and a low-pass FIR filter, an implementation using a CIC filter followed by a FIR compensation filter and at last a CIC filter followed by a bandpass filter in the ultrasound frequency range are implemented.

#### 4.4.6. SRP

The Steered Response Power (SRP) calculations take place at the last stage of the computations. Users can define the number of samples *L* to be used to compute the SRP (Equation (26)). Ideally, a power of 2 length is preferred so that arithmetic shift operations on FPGA can be used as division. Two SRP modes are supported during emulations: streaming and block mode. The former one is aimed to allow a more fine grained response where the evolution of the response can be visualized. Here, a sliding window with length *L* is used and at each input a new SRP value is computed. In the second method, a SRP value is computed every *L* samples and allows to reduce the amount of data to be transferred and visualized. This is also the preferred implementation for FPGAs. Thanks to the Parseval’s theorem, both approaches are computed in the time domain enabling streamed SRP computations.

#### 4.4.7. Commutative Computations

**Figure 5:**
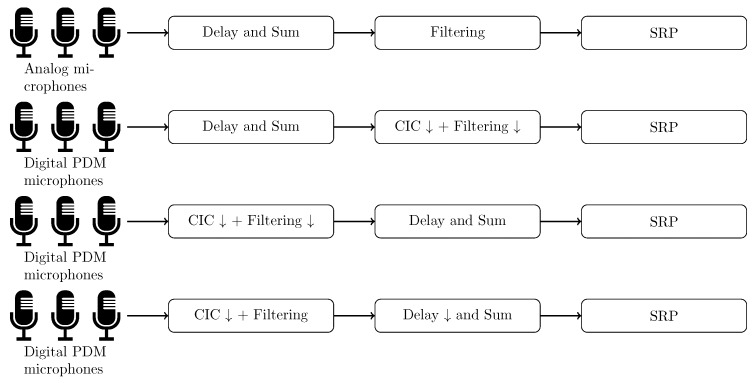
Delay and sum followed by a filtering stage for analog and digital based microphones. A “↓” denotes a decimation step.

The operations involving delay and sum, filtering and decimation have been proposed in different commutative sequences. This is especially the case for PDM microphones where the decimation and filtering can be computed prior to the D&S as well as afterwards. Moreover, a hybrid solution has been proposed by da Silva et al. [10,55]. Following beamforming sequences are currently supported by the emulator (Figure 5):

1. Delay and Sum + Filtering for analog based microphones,
2. Delay and Sum + CIC + Filtering for PDM based microphones,
3. CIC + Filtering + Delay and Sum for PDM based microphones,
4. CIC + Filtering + Delay and Decimation and Sum.

Modifying the order of operations allows to select the appropriate method where the balance between required processing capabilities and possible accuracy has to be considered. In case of the last options, the number of parallel filters is directly related to the number of available microphones, while in options 1 and 2 this number scales with the number of steering angles. In case the number of microphones *m* is smaller than the number of steering angles, strategy 3 will require more processing capabilities. However, the delay and sum process offers better localization results with a higher sampling rate. Strategy 4 has been proposed by da Silva et al. [9,10] as a hybrid solution where resource consumption on FPGA has been reduced while providing similar computation results as in 3. Implementing methods where the filtering is performed prior to the delay and sum method requires special care when computing the delay table. In Equation (20) a signal delay mechanism proper to each microphone is provided. Highest signal realignment accuracy is achieved with the highest possible sampling frequencies. First decimating and filtering the samples before realigning them will cause greater signal distortion. Therefore, an additional buffer is used at each microphone input where signals are delayed according to the signal delay property of the respective microphone.

### 4.5. Response Results Output (Step 3)

Results of each beamforming response are stored into an output directory. After computations, this directory contains the original input files utilized, i.e., sound sources, emulation file, SDP-file and microphone array. A file containing the delay tables utilized during delay and sum is also available. This file is organized as the table depicted in Figure 3 in a Comma Seperated Values (CSV) format. The SRP results are stored in a second CSV-file. Each row corresponds to a computed set of acoustic sources while each column represents a steering vector. The acoustic sources are subdivided into dynamic and static sources. The static sources can be found in the sound sources file. The dynamic sound source however can differ from the frequency and or position as described in the sound source file. To allow coherent retrieval of each “response–acoustic” source property, the parameters used to compute each response against this source are stored in an additional file. This file contains the type of source, the frequency and the position. For each of the output files an additional Matlab compatible file is created for easy processing in Matlab. The other files are intended for the CABE client and contain additional information regarding file organization. Aside from the response output files, the emulator also generates a project file containing a link to all other files. This file is processed by the CABE client application and also contains the eventual error codes occurred during processing as well as the time of processing, the CPU model used, the number of computing cores and the amount of RAM memory on which the emulation has been performed. This serves as statistics in order to predict the amount of processing time a new emulation would take. The project is generated following the YAML file format.

### 4.6. Metrics Generator (Step 4)

The metrics generator computes the proposed metrics so that the beamforming performances of a given microphone array can be evaluated and are implemented in an independent fashion so that the beamforming results can be re-evaluated when new metrics are added. This especially allows to (re-)evaluate complex microphone arrays which require long processing times in a faster way and to avoid duplicate computations.

The results of the metrics are stored in a dedicated subfolder of the output folder. All computed metrics are linked into the output project file of the computed beamforming.

### 4.7. HDL Package Generator (Step 5)

The provided beamforming methods consist of well known building blocks such as a delay and sum module, CIC filters, FIR filters, halfband filters, etc. Aside of a few parameters such as the tap coefficients of the filters, the order of the CIC filter, the amount of microphones, the delay tables and the order of computations, these blocks can be approached as generic building modules. Therefore, all modules are wrapped into a module generator that defines the required parameters for each block. At a higher level, all modules are interconnected following the configuration of the required processing flow of the microphone array, which is extracted from the microphone array processing emulation data structures. The output is stored into a separate folder containing only the VHDL code. In this research we primarily target the Zynq based platforms from Xilinx since these contain both a programmable and processing logic [56] facilitating in situ processing of the beamforming information on the local processor [10]. Currently, only the “CIC + Filtering + D&S for PDM based microphones” architecture can be generated. The other methods, due to the possible high number of steering vectors, need advanced data flow scheduling schemes which are currently not automated by the tool.

### 4.8. User Web Interface Database and Task Manager

The User Web Interface acts as a proxy between the client application and the emulators running in the background. The User Web Interface is written in PHP, where GET and POST requests can be issued in order to retrieve or add information into the MySQL database. The web interface comprises 4 major parts:

- the database from which the status of each of the emulations can be followed,
- the module which enables the client application to retrieve the necessary configurations and constraints for generating proper emulation files,
- the Task Manager enables to schedule emulations and to communicate with the emulator computers in the back-end and,
- a module keeping track of the required processing time per type of machine and per type of emulation request.

The communication between the client application and webserver is performed by means of RESTful API calls. Configuration files for the client application are generated in the YAML format in the webserver following the content of the database. The issued emulations per user and the settings for the several available processing stages are transferred following this pattern.

The database keeps track of the emulations which have to be launched on the back-end computers. Therefore, small PHP scripts allow an idle back-end computer to POST a request for queued emulations. If an emulation is still queued for processing, it will be transferred to this idle emulation computer. The status of this emulation is changed to “being processed” via a mutex to avoid redundant computation on multiple computers.

A last part of the interface serves to estimate the amount of time required to perform a given beamforming. This is especially the case when running large emulations with many steering vectors.

### 4.9. CABE Client

**Figure 6:**
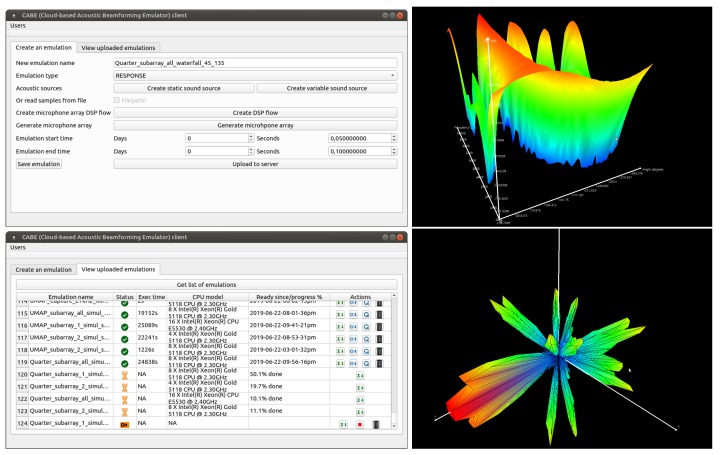
CABE client application. On the Left Top side users can create and upload new emulations to the User Web Interface. On the Left Bottom side, a list of emulations can be retrieved. Emulations which are ready can be visualized can be plotted with the available plotting tool. On the Right Top a waterfall diagram and on the Right Bottom side a 3D polar plot are shown.

In the first part, users utilize the CABE client application (Figure 6) which automates the generation of the required input files for the emulator and uploads the parameters to the webserver. This application also downloads the available information regarding the filtering and beamforming chains that can be selected. Once a complete emulation has been set up, users can archive the configurations and upload the complete emulation to the webserver. Once uploaded, users can follow the progress of the emulations in a second tab. Several options are available, including the possibility to download the uploaded emulations, to request to queue the emulation for processing, to download the beamforming results and to (re-)compute metrics. When the results are available, users can visualize the beamforming results with a dedicated graphing tool. All results are stored into the webserver’s database so that users can retrieve them on any computer.

In the second part, the client uses the information from the obtained emulation to generate appropriate graphs. Depending on the type of steering method utilized, a Cartesian or a spherical graph is generated. In case that a 2D steering mechanism is used, a Waterfall diagram is generated. The SRP is plotted as a function of the steering angle and the frequency and the ability is provided to move the camera (view point) anywhere around the graph. From the waterfall diagram, it is also possible to request a polar plot for a specific frequency. Choosing the minimum and maximum values for the frequency, steering angle and SRP values is also possible. All the graphs are generated with the OpenGL library [57] which is freely available. All graphs can be exported to a Portable Document Format (PDF) file. This is done using the open source library of gl2ps [58].

If 3D steering vectors are used, a spherical graph is generated. All the vectors composing the steering vector space are first sorted out via a triangle splitting algorithm in non overlapping triangles. Since the algorithm has a complexity of $O(n^{2})$ and to increase plotting speed, the obtained triangles are stored into a CSV-file for reuse when the same graphs are regenerated. This graph does offer clipping planes. Exporting the graphs to a PDF-file is possible and is done in a similar fashion as for Waterfall diagrams.

## 5. Demonstration of the CABE Platform

The cloud based emulator is capable of computing a beamforming for any given microphone following several steering methods. In this section, we demonstrate and evaluate the computed results of two different microphone arrays. Both arrays have also been manufactured in order to validate the platform, as will be explained in Section 6. The arrays evaluated consist of the ultrasound PDM enabled SPH0641LU4H microphones [42]. These microphones operate in a sampling frequency range between 1 MHz up to 4.8 MHz and are capable of capturing acoustic waves up to 80 kHz.

**Figure 7:**
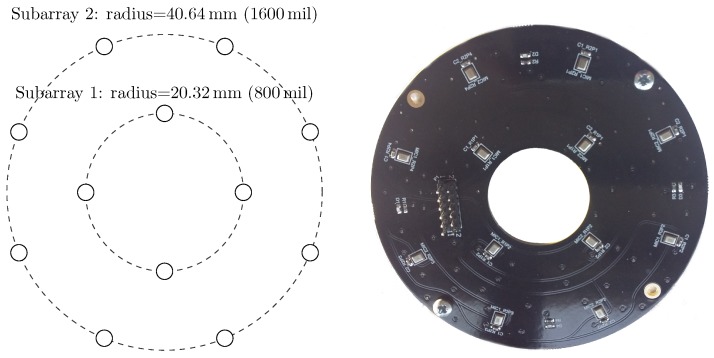
Microphone locations on the UMAP array (**Left**). The outer subarray consists of 8 microphones which have an offset of $22.5^{\circ }$ to the axis. The 4 inner microphones are all located on 20.32 mm from the center. The PCB of the array allows to mount an additional camera or speaker for several applications (**Right**).

**Figure 8:**
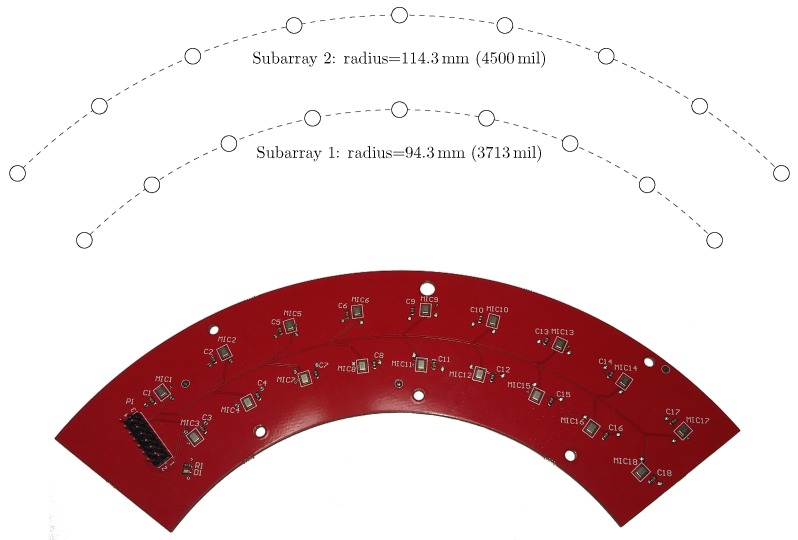
The Quarter array consisting of 18 microphones (**Top**). The most outer microphones describe an aperture angle of $90^{\circ }$ around the vertical axis. The PCB of this array (**Bottom**) enables to mount additional devices at the center of arcs describing the placement of the microphones.

1. The first array—the Ultrasonic Multiple Access Positioning (UMAP) array—to be evaluated consists of 12 microphones placed in 2 rings, see diagram in Figure 7. The primary purpose of this array is to evaluate capability of finding an ultrasound source in given AoA. The inner subarray—subarray 1—is composed of 4 microphones located on a radius of 20.32 mm from the center. The outer subarray is composed of the 8 remaining microphones where all microphones are located on a radius of 40.64 mm from the center. Although not provided in the emulation file, the Printed Circuit Board (PCB) provides a hole in the center (Figure 7 Right) so that a speaker or camera can be mounted for additional experiments.
2. The second array—the Quarter array—used in the experiments consists of 18 microphones placed in 2 arcs and is shown in Figure 8. This array is designed to help visually impaired people and is mounted on the front head of the person. A transducer mounted on top of the array emits an ultrasound pulse which is reflected by nearby located objects. Measuring acoustic information coming from the back is not desired. Therefore, the array is designed to steer in an aperture angle of a quarter circle, i.e., $90^{\circ }$, in the direction of the convex side. This array consists of 2 subarrays with 9 microphones each. The outer subarray has a radius of 114.3 mm while the inner subarray is arched at 94.3 mm. The shape of the array allows to have a limited number of microphones when covering a limited amount of the steering aperture.

For both arrays, a 4-layer PCB-board is used together with the necessary via-stitching and carefully layed tracks in order to avoid signal degradation from the PDM microphones.

### 5.1. Frequency Response from a Single Acoustic Emitting Position

Evaluating the performance of the proposed microphone arrays is done in several stages. In a first stage, the performance of the array is evaluated by computing the waterfall diagram over the available frequency range of the microphones. This waterfall diagram is computed while utilizing the “Equalpolar” steering distribution. Along with the waterfall diagram, the performance metrics describing the performance of the arrays over the complete frequency range are computed. During this experiment the performance of the arrays while disabling subarrays is evaluated. In all experiments, a single acoustic sound source located at 5 m distance from the array at an angle of $180^{\circ }$ and $90^{\circ }$ for respectively the UMAP array and the Quarter array is used. The UMAP array is evaluated in $360^{\circ }$ with 128 steering orientations while the second array is evaluated between $45^{\circ }$ and $135^{\circ }$ with 64 steering orientations. The results are computed by applying a monochromatic sound source with frequencies ranging from 100 Hz to 80 kHz in steps of 100 Hz. The PDM frequency is set to 4.8 MHz so that a maximum allowed acoustic frequency of 80 kHz can be captured. The experiments are carried out with the beamforming method where the acoustic PDM signals are first filtered and decimated before being delayed and summed. The filtering and decimation sequence is as follows:

1. CIC filtering: decimation factor of 15 with a differential delay of 2.
2. FIR filter: A compensation filter of 64 taps where the effects of the CIC filter and the microphone characteristics are flattened within a margin of 2 dB in a frequency range between 1 and 65 kHz.
3. Halfband filter 2: A filter of order 32 with a cutoff frequency set to 80 kHz.
4. A last decimation step which decimates with a factor of 2.

The input signals from each of the microphones is therefore decimated by a factor 30, resulting in an output sampling frequency of 160 kHz which is used to generate the delay tables for the D&S beamforming method.

**Figure 9:**
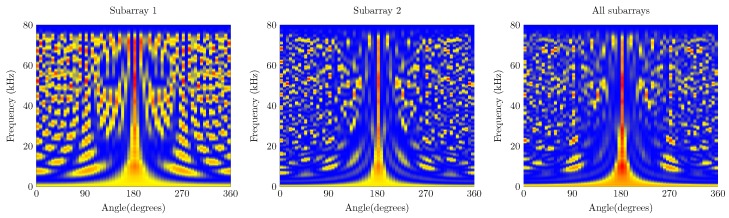
Waterfall diagram of the UMAP array. When only the 4 inner microphones are enabled, the diagram shows a lot of side and grating lobes (**Left**). These lobes are attenuated when using the outer 8 microphones (**Middle**). The (**Right**) graph shows the best waterfall diagram for this array when all microphones are enabled. In all cases, a monochromatic sound source with increasing frequency is positioned at a distance of 5 m at $180^{\circ }$.

**Figure 10:**
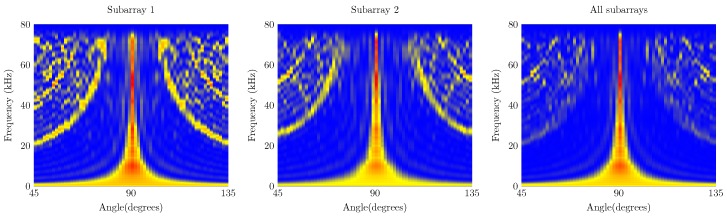
Waterfall diagram of the Quarter array. When only the 9 inner microphones are enabled (subarray 1), the diagram shows a lot of side and grating lobes (**Left**). These lobes are attenuated when using the outer 9 microphones (**Middle**). The (**Right**) graph shows the best waterfall diagram for this array when all microphones are enabled. In all cases, a monochromatic sound source with increasing frequency is positioned at a distance of 5 m at $90^{\circ }$.

The waterfall diagrams of both arrays are shown in Figure 9 and Figure 10. The effects of disabling the subbarays in the microphone array results in a lower probability of finding a sound source in a given AoA. In case of the UMAP array, when only the inner subarray is enabled, the resulting waterfall diagram shows several grating and side lobes. The regular pattern of these lobes is due to the spacing of the 4 inner microphones which are placed in a square enhancing spatial aliasing. To mitigate the effects, the outer 8 microphones are placed in a circular pattern. When all microphones are enabled, the beamforming results in a higher probability to find the AoA of a given sound source. The waterfall diagram of the Quarter array shows a higher probability of finding a sound source at $90^{\circ }$ than the UMAP array, for all combinations of enabled subarrays. This is mainly due to utilizing more microphones in a larger array. The waterfall diagrams allow to visually assess the beamforming quality. However, describing the quality of beamforming by means of the metrics enables to identify the frequencies on which the array would result in the highest probability of finding a sound source.

**Figure 11:**
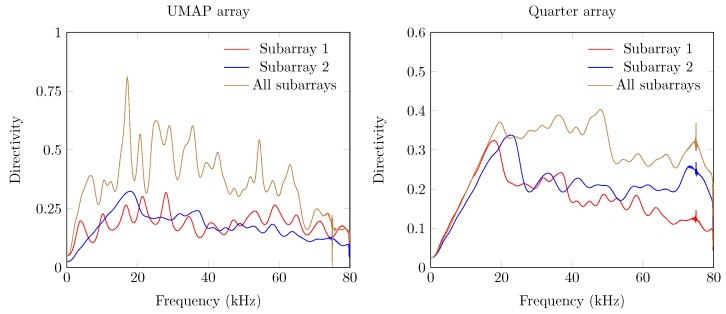
Directivity index of the UMAP array (**Left**) and the Quarter array (**Right**). Higher values are preferred. The UMAP array shows local optima describing higher probability to find a sound source for that particular frequency.

The first metric to be described is the directivity index of the UMAP and the Quarter arrays which are shown in Figure 11. For both arrays, the directivity is computed at the supposed AoA of the acoustic source. In case of the UMAP array, this metric peaks (when all microphones are enabled) at approximately 17.1 kHz and describes other peaks around 25, 35 and 55 kHz. The Quarter array shows a more regular directivity between 20 and 48 kHz.

**Figure 12:**
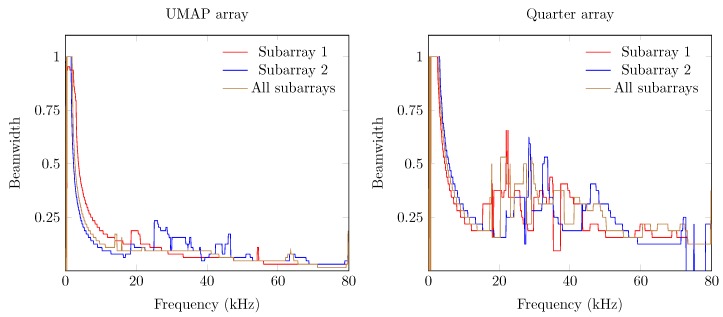
Beamwidth of the UMAP array (**Left**) and the Quarter array (**Right**). Lower values indicate thinner main lobe at this orientation. The beamwidth generally decreases while the frequency increases. One should note that the beamwidth of the Quarter array is computed over an area 4 times smaller than for the UMAP array, resulting in values approximately 4 times larger than when computed for a complete circle. Although one may expect a fluent decreasing trend, the peaks appearing beyond 20 kHz result from the treshold function including the second smaller lobe into the computations.

The second metric described here is the beamwidth and describes the width of the main lobe. A thinner main lobe results in a higher ability to discriminate multiple sound sources from each other. This latter bounded to the main lobes and not to the grating and side lobes. Figure 12 depicts the beamwidth for the UMAP and the Quarter array. In general, the beamwidth decreases with increasing frequency. The beamwidth here is depicted as a relative metric and is lower for the UMAP array than for the Quarter array.

**Figure 13:**
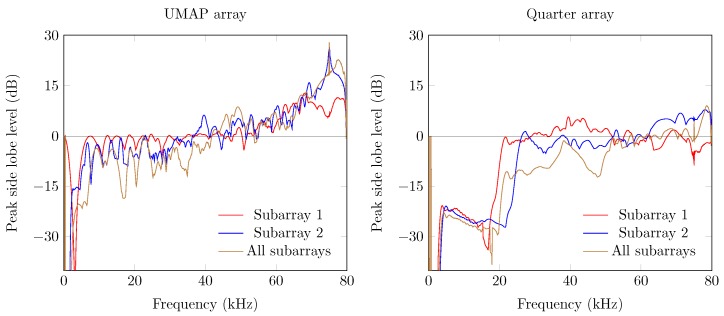
PSLL values of the UMAP array (**Left**) and the Quarter array (**Right**). Local minima can be observed for the UMAP array when all subarrays are enabled. Positive values indicate that the current supposed main beam is superseeded by another peak.

The directivity and the beamwidth metric describe how a supposed main beam performs compared to the complete area of interest of the microphone array. However, in case one utilizes small arrays, the location of the supposed AoA may not show the main peak of the beamforming result. The PSLL allows to confirm if the supposed main beam corresponds to the detected main beam. Figure 13 shows the PSLL of the UMAP and the Quarter array. Here a lower negative value of the PSLL offers a higher probability to find the AoA of the sound source. Positive PSLL values indicate that the beamforming of the array detects another AoA than the supposed sound source locations. When all subarrays are enabled the PSLL values of the UMAP array allows to detect the main beam up to approximately 42 kHz. From this point, the PSLL values are positive with an exception at 54.4 kHz. The Quarter array shows lower PSLL values up to 20 kHz. When all subarrays are enabled, the main peak can be found up to a frequency of approximately 55 kHz. Local minima in both graphs can be correlated with the directivity where a local optimum is observed at the same frequencies.

**Figure 14:**
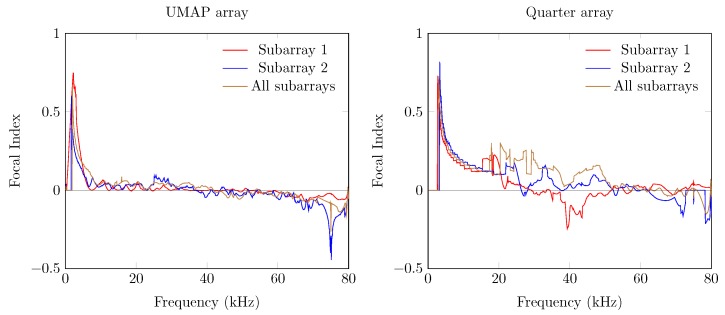
FI of the UMAP array (**Left**) and the Quarter array (**Right**). In case of the UMAP array, the FI rapidly drops to a minimum and crosses the Abscis at 42 kHz (all subarrays enabled). The Quarter array shows a better FI which crosses the Abscis at approximately 55 kHz (all subarrays enabled).

The last metric—the FI—combines the beam pattern values outside the main beam along with the linearized PSLL values of the supposed main beam. A value between 0 and 1 indicates that the main beam could be found at the current expected steering angle. Negative values relate to the PSLL values and indicate that the current steering angle does not converge to the main beam observed by the microphone array. In case of the UMAP array, positive values can be found up to 42 kHz while the Quarter array ranges up to 55 kHz (Figure 14).

From the above mentioned metrics, a few frequencies can be chosen so that beamforming would result in optimal detection of AoA. The conditions to be met are:

- Optimal (higher) values for the directivity,
- The lowest possible beamwidth,
- The lowest possible (negative) values for PSLL and,
- Highest (positive) values for FI.

**Figure 15:**
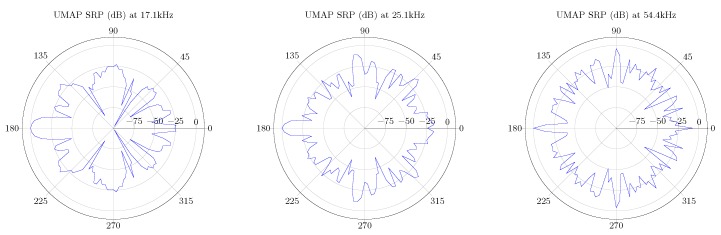
Polar plots of the UMAP array at 17.1 kHz (**Left**), 25.1 kHz (**Middle**) and 54.4 kHz (**Right**). These plots are bound between $0^{\circ }$ and $360^{\circ }$ and show the main lobe at $180^{\circ }$. The surrounding lobes have a value of approximately −12 dB for 25.1 kHz and 54.4 kHz. At 17.1 kHz, the sidelobes have values around −20 dB describing a higher probability of finding a sound source of that particular frequency at $180^{\circ }$.

**Figure 16:**
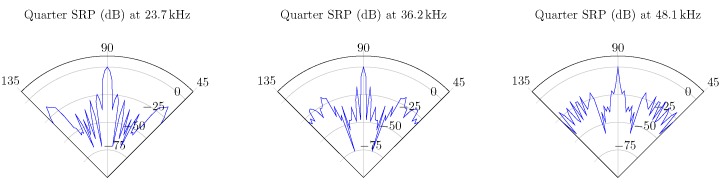
Polar plots of the Quarter array at 23.7 kHz (**Left**), 36.2 kHz (**Middle**) and 48.1 kHz (**Right**). These plots are bound between $45^{\circ }$ and $135^{\circ }$ and show the main lobe at $90^{\circ }$. The other lobes have a value of approximately −18 dB and confirm the results obtained from the metrics.

In case of the UMAP array, some of the optimal frequencies at which a sound source can be found at $180^{\circ }$ are 17.1, 25.1 and 54.4 kHz. For the Quarter array the selected frequencies are 23.7, 36.2 and 48.1 kHz. In the latter case, the sound source is found at an angle of $90^{\circ }$. Based on the metrics, other frequencies can also be selected. In Figure 15 the polar plots of the selected frequencies for the UMAP array are depicted. In Figure 16 the polar plots of the selected frequencies for the Quarter array are shown. The polar plots of the Quarter array are bound between $45^{\circ }$ and $135^{\circ }$, which correspond to the steering orientations used during the computations.

### 5.2. Acoustic Source Emitting from Multiple Positions

**Figure 17:**
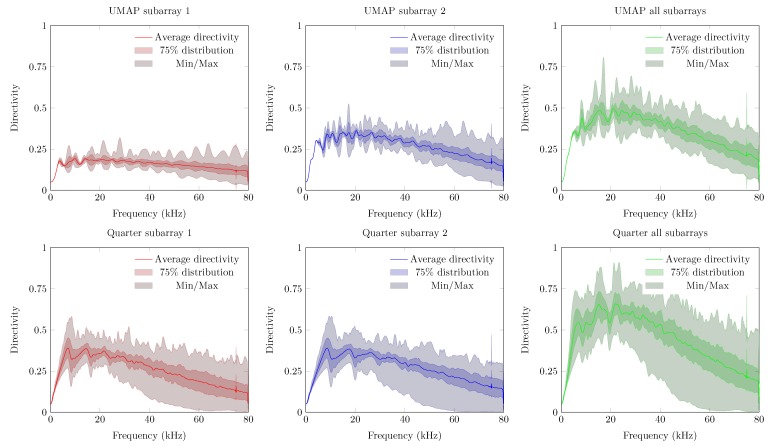
Directivity on multiple positions. The directivity of the UMAP array (**Top**) and the Quarter array (**Bottom**) when the inner (**Left**), outer (**Middle**) and both (**Right**) subarrays are enabled.

In Section 5.1 the results of the microphone arrays when subjected to a single monochromatic sound source with increasing frequency from one position are demonstrated. However, sound sources can be placed at any position around the microphone array. To estimate the response of the proposed microphone arrays, a single acoustic source emitting monochromatic waves from different positions around the microphone arrays is used. From here, the different metrics can be computed against each emitting position. For each frequency, the minimum, maximum, average and the 75 percentiles (Q3 factor) of each metric are computed. To obtain these steered metrics, the response of the microphone array is sampled with a limited number of emitting positions. The obtained minimum, maximum, average and Q3 values of each steered metric are representative in case a sufficient number of equally distributed positions over the area of interest. The steered metrics are computed for both arrays with the different combination of subarrays enabled. For both arrays we compute the steered metrics in a horizontal plane for the full $360^{\circ }$. The computations are done at 32 evenly distributed angular positions in the $360^{\circ }$ horizontal plane at a radius of 5 m from the microphone array. At each position, the acoustic source emits signals between 100 Hz up to 80 kHz with a frequency interval of 100 Hz. The steered directivity plots for both arrays are shown in Figure 17.

The average directivity for both arrays increases while the number of microphones utilized increases. The directivity increases for both arrays and for all combinations of subarrays up to a given threshold frequency, beyond which a directivity decrease can be observed. A noticeable difference between both arrays can be found in both the level of average directivity and the range between the minimum and maximum directivity. The UMAP array generally offers a lower average directivity. However, the range between the minimum and maximum directivity (and the Q3 factor) is much lower compared to the results obtained from the Quarter array. The circular pattern of the UMAP array allows to steer in all orientations with a lower difference in SRP values. The Quarter array has specifically been designed to match the response of a quarter of an array resulting in a lower SRP performance around $0^{\circ }$ and $180^{\circ }$.

**Figure 18:**
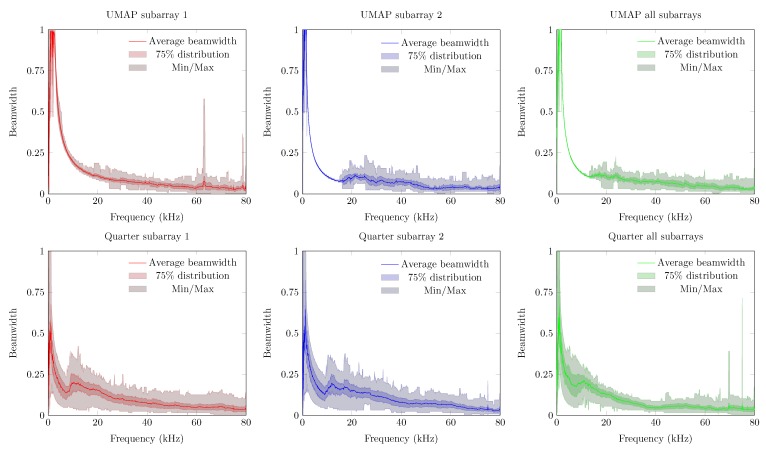
Beamwidth on multiple positions. The beamwidth of the UMAP array (**Top**) and the Quarter array (**Bottom**) are shown with the inner (**Left**), outer (**Middle**) and all subarrays (**Right**) enabled.

The obtained beamwidth also varies depending on the shape of the microphone array. This is shown in Figure 18. In the experiments, the size and amount of microphones do not necessarily induce a smaller beamwidth. The average steered beamwidth of the Quarter array in all orientations is slightly higher compared to the UMAP array. In case of the Quarter array, the descend rate of the beamwidth at lower frequencies is more pronounced than for the UMAP array. However, even though the Quarter contains more microphones in a bigger shape, the variability of the beamwidth shows that this array is less suited for omnidirectional measurements compared to the UMAP array. At higher frequencies, 25 kHz and beyond, both arrays show a similar performance. This is especially the case when all subarrays are utilized.

**Figure 19:**
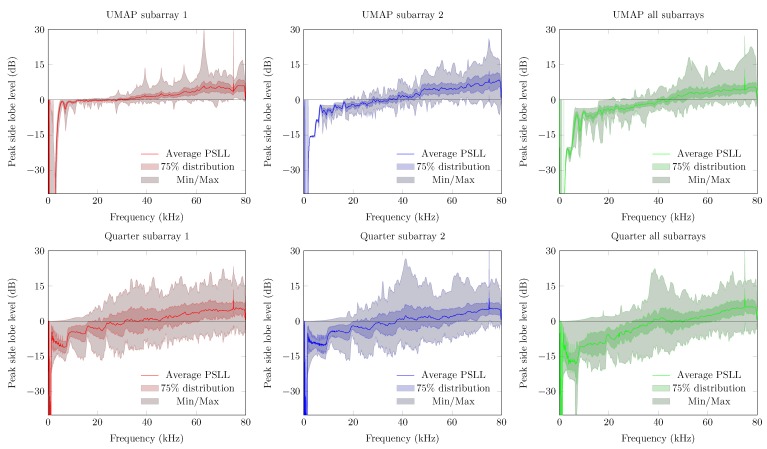
Peak side lobe level on multiple positions. The peak side lobe level of the UMAP array (**Top**) and the Quarter array (**Bottom**) are shown with the inner (**Left**), outer (**Middle**) and all subarrays (**Right**) enabled.

The PSLL metric allows to determine whether a given microphone array detects the AoA of a sound source at the expected angle. For this metric the average, the minimum and maximum, and the Q3 factor of the PSLL are computed. Positive PSLL values indicate that a higher peak at another angle overwhelms the expected AoA. The steered PSLL values of both arrays are shown in Figure 19. The UMAP array shows, as in Section 5.1, higher values for the average PSLL compared to the Quarter array. However, a comparable study regarding the variability on the metric can be observed. In case of the Quarter array, the minimum and maximum of this metric reaches 0 dB at very low frequencies. This threshold is only reached at a frequency of approximately 16 kHz for the UMAP array when utilizing all subarrays. The Q3 distribution however, reaches the 0 dB only at 28 and 36 kHz for respectively the Quarter and UMAP array when all subarrays are enabled. The latter indicates that the AoA of an acoustic source can be found in 75% of the cases up to these frequencies.

**Figure 20:**
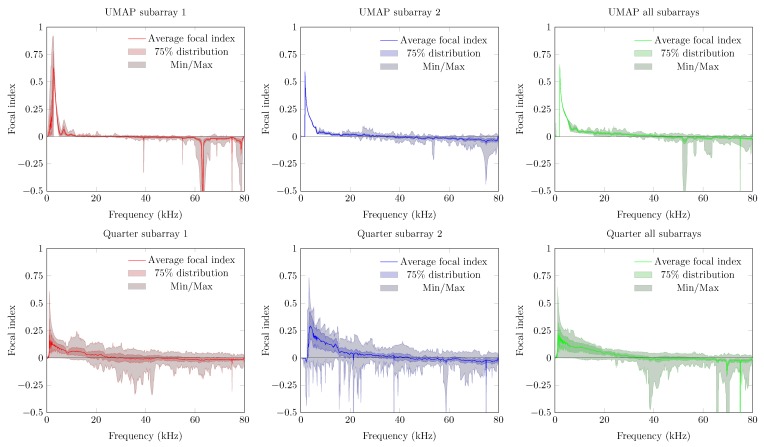
FI on multiple positions of the UMAP array (**Top**) and the Quarter array (**Bottom**). Here, the graph represents the results when subarray 1 (**Left**), subarray2 (**Middle**) and all subarrays (**Right**) are enabled.

The last metric to be discussed here is the steered FI (Figure 20). Negative values of focal indices indicate the main beam could not be found at the expected AoA of the acoustic source. Better average results are again obtained with the Quarter array. Similarly to the other metrics, the variability on this metric is more pronounced for the Quarter array, resulting in zero or slightly negative values at lower frequencies. In case that all subarrays are enabled on the UMAP microphone array, negative focal indices can be found beyond 16 kHz. However, the Q3 factor of the Quarter array reaches the abscis at approximately 28 kHz. The UMAP reaches this point at approximately 38 kHz, indicating that in all steering orientations the array is capable of finding the supposed AoA of the acoustic source in 75% of the cases.

**Figure 21:**
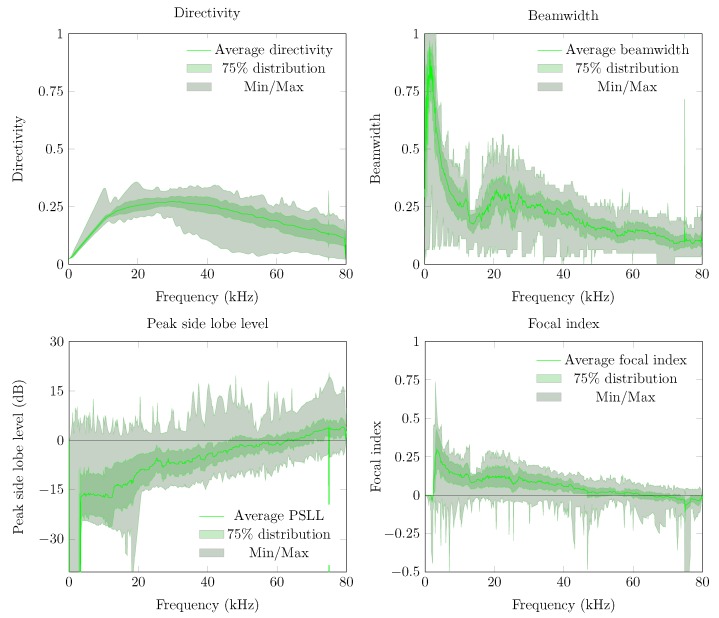
Metrics on multiple positions of the Quarter array between $45^{\circ }$ and $135^{\circ }$. The directivity (**Top left**), the beamwidth (**Top right**), the PSLL (**Bottom left**) and the FI **(Bottom right**) are shown. The graphs represent the results of the Quarter array when all subarrays are enabled and the position of the acoustic source varies between $45^{\circ }$ to $135^{\circ }$.

The Quarter array offers a result which is not optimized for locating an acoustic source in $360^{\circ }$. Therefore, in the following experiment the steering vectors are bounded between $45^{\circ }$ and $135^{\circ }$. The results are shown in Figure 21. The directivity of the Quarter array between $45^{\circ }$ to $135^{\circ }$ shows a lower average value. The Q3 factor (and the minimum and maximum) shows a smaller deviation around the average indicating that the array performs more consistently in this region of interest. The beamwidth however generally increases with a factor of four compared to the previous emulated steered beamwidth. This is due to a reduction of the area of interest by a factor 4. More importantly, the average PSLL factor decreases whereas the Q3 factor of the PSLL crosses the Abscis at approximately 45 kHz. The minimum and maximum boundary however indicate that the array does not perform better than when locating a sound source in all directions. The latter is reflected in the FI, where the Q3 factor crosses the Abscis around 45 kHz, but the minimum and maximum observed values do not allow to always detect a sound source unambiguously in the given area of interest.

### 5.3. 3D Polar Plot

**Figure 22:**
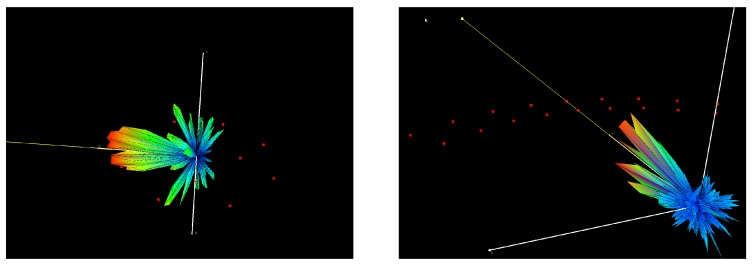
Polar plot in 3 dimensions of the UMAP array (**Left**) and the Quarter array (**Right**). The plot is obtained at a frequency of 17.1 kHz for the UMAP array and at 21.5 kHz for the Quarter array. The AoA of the sound source is highlighted with the yellow line. The shape of the microphone array is represented with red points in the graph.

**Figure 23:**
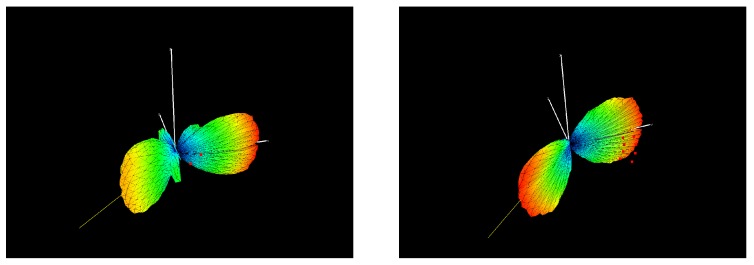
Polar plot in 3 dimensions of the UMAP array (**Left**) and the Quarter array (**Right**) with two emulated sound sources located at [0,5 m,0] and at [0,−5 m,−5 m]. In case of the Quarter array, the sound sources emit at a frequency of 8 kHz and 8.8 kHz. The UMAP array is pointing to sources with a frequency of 8 and 6.2 kHz.

A similar plot as the polar plot in 2D can be obtained when steering in 3 dimensions. In this experiment a 3D beamforming of both microphone arrays is obtained by utilizing the same delay and sum beamforming and filtering approach as described in Section 5.1. The 3D plots are shown in Figure 22 and Figure 23 and describe a symmetrical polar plot around the XY-plane. This effect is due to the planar configuration of both arrays. The main lobe for both arrays points to the direction of the sound source.

### 5.4. Beamforming Using Fixed Point Precision DSP

**Figure 24:**
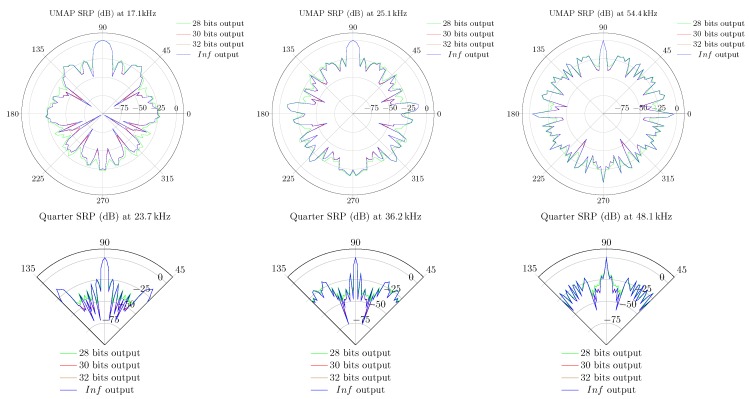
SRP error due to the output bitwidth of the the UMAP array (**Top**) and the Quarter array (**Bottom**). The UMAP array is evaluated at frequencies 17.1, 25.1 and 54.4 kHz while the UMAP array is evaluated at 23.7, 36.2 and 48.1 kHz. All results are normalized to the highest observed SRP value.

The emulations performed in Section 5.1 are obtained by using double precision arithmetic. FPGAs and other low power embedded systems may compute the results using fixed point and or integer arithmetics. The emulator also allows to estimate the error $\xi^{\prime }\left(\vec{u},S,z\right)$ between a given amount of bits resolution and double precision. In the experiments the SRP of both arrays when all subarrays are enabled is computed. In both cases, an acoustic source is placed at an angle of $90^{\circ }$ relative to the microphone arrays. The SRP in double precision format is computed as a reference. Thereafter the same SRP with a limited number of bits in fixed point format is computed, where 18 bit coefficients for the filters are used. The output bitwidth used in the experiments ranges from 28 up to 32 bits. Lower bitwidths could not be used since these result in a zero SRP. Higher bitwidths result in a SRP which nears the SRP in double precision format, and thus resulting in a lower $\xi^{\prime }\left(\vec{u},S,z\right)$ error. The SRP of the UMAP array is computed at 17.1, 25.1 and 54.4 kHz, while the Quarter array is evaluated at 23.7, 36.2 and 48.1 kHz. The results are shown in Figure 24. The double precision SRP is represented by the “Inf” legend. All SRPs are normalized to the maximum observed value. In all cases, a lower number of output bits would result in a more flat SRP response. In case 28 output bits are used, the logarithmic error $20\cdot log\left(\xi^{\prime }\left(\vec{u},S,z\right)\right)$ attains almost 25 dB at some points of the SRP. When 30 bits are used, the SRP matches more closely the expected SRP while at 32 bits the SRP differences with the double precision output are almost not noticeable.

### 5.5. Compute-Time of the Emulations

The emulations mentioned in the above paragraphs have been computed on 4 server machines. Depending on the requested operations and the machines on which the operations are performed, the computations take a given amount of time. The configurations of the machines are listed in Table 1. The different requested operations with the time required to obtain the results are listed in Table 2. The machine on which the operations have been computed is also listed. During the computations all available cores in each machine are used.

## 6. Validation of the Emulation Platform

**Figure 25:**
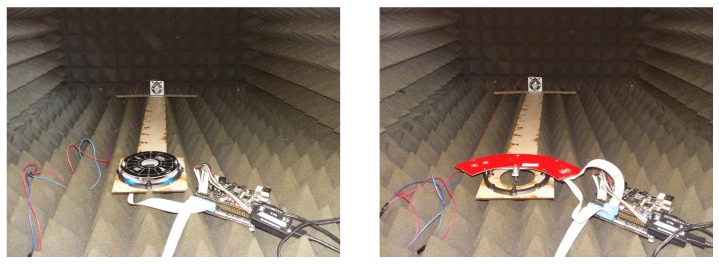
Setup of the microphone arrays in the anechoic box, with the UMAP array (**Left**) and the Quarter array (**Right**). Both arrays are mounted onto a ruler. At the other end of the ruler a piezoelectric transmitter emits acoustic waves at a distance of 60 cm of the microphone arrays. The FPGA is the device at the front right in the box with a UART connection with a computer.

**Figure 26:**
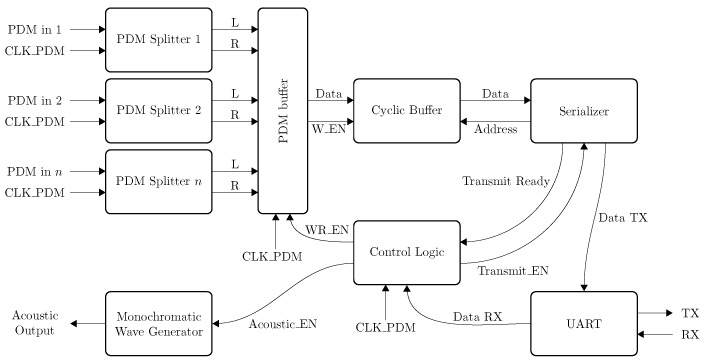
FPGA logic of the measurement setup. The link between the computer and the FPGA is done via the UART connection (TX and RX). When the control logic receives the command to start capturing samples, the monochromatic wave generator emits an acoustic wave with the appropriate frequency. The microphones send data to the FPGA via the PDM signals, which are splitted in left (‘L’) and right (‘R’) channels. The PDM buffer allows to synchronize the left and right channels before the samples are stored into the cyclic buffer. After capturing, the serializer transfers the samples as data of 1 byte via the UART to the computer. The PDM_CLK runs at 4.761 MHz.

The last part of the experiments consists of validating the emulation platform by computing beamforming results of acoustic captured samples. To mimic an acoustic reverberation free and open field environment, measurements are performed in the anechoic boxes built by Carvalho et al. [59]. These boxes offer enough space to fit a complete test setup which consists of a microphone array, a speaker and an FPGA attached to both the speaker and the microphone array. The complete setup in the anechoic box is depicted in Figure 25. To evaluate the response of the array on acoustic sources emitting from different angles, a ruler with a fixed length of 60 cm with the possibility of mounting the array and the speaker with angular intervals of $2.81^{\circ }$ is lasercut. The distance of 60 cm allows to fit the complete setup in the anechoic box and to perform measurements which can be considered far-field. In the experiments a 250ST180 piezo electric transducer with a resonance frequency of 25.5 kHz [60] is used. Although this emitter offers a limited acoustic bandwidth of approximately 2 kHz, we noticed in the previous section that the optimum capturing frequency range of both arrays is situated around this resonance frequency. The FPGA allows to send acoustic monochromatic waves with a known frequency while receiving the PDM signals from the microphones at the same time. The FPGA board is a Zynq based platform manufactured by MYIRtech [61]. This board comes with a Zynq7020 FPGA running at 100 MHz and offers 3 Peripheral Module interface (PMOD) connectors through the IO-cape board [62] allowing easy connection with the Quarter and UMAP microphone array boards. The FPGA design which captures and generates the acoustic waves is shown in Figure 26. Capturing the PDM samples from the microphones is done in 3 steps.

1. At first, a clock signal is generated to drive the microphones. The clock the microphones is set at the highest possible clock speed of 4.761 MHz possible (i.e., with a clock divider ratio of 21).
2. Secondly, the PDM signals are captured from the microphones. Two microphones share the same PDM multiplexed data line. To retrieve the individual signal from each microphone, this signal is demultiplexed in the FPGA in a ‘left’ and ‘right’ channel by the PDM splitter modules.
3. During the last step, the PDM values are stored in a cyclic buffer before being transferred to a computer for later processing. This latter is done via a Universal Asynchronous Receive Transmit (UART) link between the FPGA and a computer.

A state machine ensures the proper sampling of the microphones and to transfer the samples in the appropriate order to the computer.

We perform a microphone calibration routine so that the individual $T_{delay}$ (see Section 2.4) of each of the microphones can be obtained. This calibration setup consists of a transmitter emitting an ultrasound wideband signal which is captured by the individual microphones in the array. The signals of the microphones are correlated with a reference microphone arbitrarily chosen in the array. Each $T_{delay}$ is obtained by comparing the expected time of the correlation peak with the obtained time of the correlation peak. The lowest $T_{delay}$ is subtracted from all $T_{delay}$ so that positive time delays are used in the delaying buffers. These time delays are taken into account during the processing of the PDM signals in the emulator.

### 6.1. Validation of the Emulated Microphone Arrays

#### 6.1.1. UMAP Array

**Figure 27:**
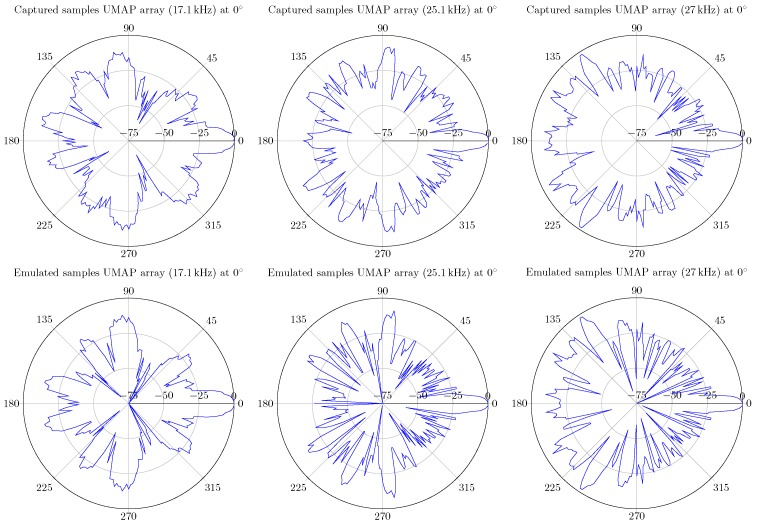
Evaluation of the SRP results of the UMAP array with captured data (Top row) and from an emulated sound source (**Bottom row**) at 17.1, 25.1 and 27 kHz. In all cases, the sound source is located at a distance of 60 cm and at $0^{\circ }$ from the microphone array.

The UMAP array is evaluated at 17.1, 25.1 and 27 kHz. All evaluations are performed using the filter + D&S architecture with a sampling frequency set at 4.761 MHz. During the capturing process, 250 k samples of each microphone (i.e., approximately 50 ms) are processed. The measured data is compared with emulated data and the results are shown in Figure 27. Although the resulting SRP values are not identical, a high degree of similarity can be noticed. This is especially the case for the major lobes, including the main lobe with a similar dB-level.

#### 6.1.2. Quarter Array

**Figure 28:**
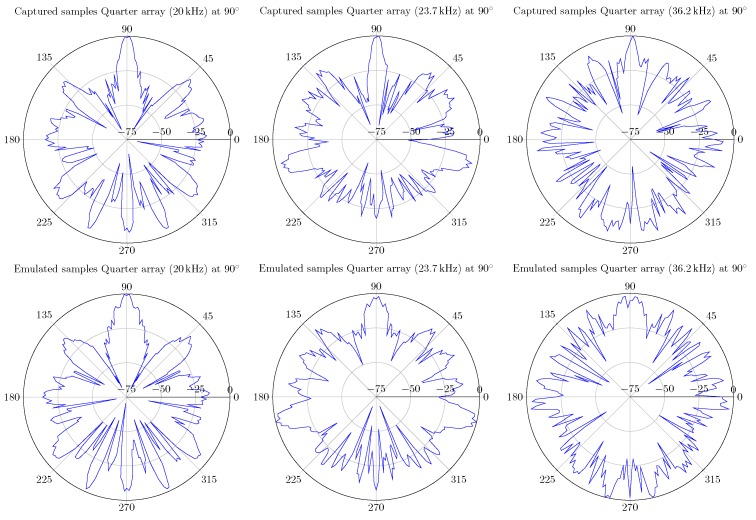
Evaluation of the SRP results of the Quarter array with captured data (Top row) and from emulated sound source (Bottom row) at 20, 23.7 and 36.2 kHz. In all cases, the sound source is located at a distance of 60 cm and at $90^{\circ }$ from the microphone array.

In case of the Quarter array, the array is evaluated at 20, 23.7 and 36.2 kHz. Evaluating the array at a frequency of 48.1 kHz would cause possible false comparisons since this frequency is well beyond the specifications of the emitting transducer. All evaluations are performed using the filter + D&S architecture with a sampling frequency set at 4.761 MHz. During the capturing process, 200 k samples instead of 250 k samples of each microphone are taken into account. This limitation of the number of samples is due to the available amount of blockram in the FPGA and the increased number of microphones. This corresponds to approximately to 40 ms of samples. The measured data is compared with emulated data and the results are shown in Figure 28. Also here, the resulting SRP values are not identical but a high degree of similarity can be noticed. This is especially the case for the major lobes, including the main lobe with a similar dB-level.

### 6.2. Compute-Time

The time required to process a file and a full emulation is also listed here. The same servers as in the previous Section are used during this process. The compute-times of the different emulations and file processing requests are listed in Table 3. Note that during these computations only one core of each machine is used. In all cases a time frame of 40 ms for both the captured samples and the emulated sound sources is taken into account during the processing. The compute-time with a captured data is generally shorter than the time required to compute with emulated sound sources. This is due to skipping the steps of emulating the sound sources and to capture within the microphones themselves. This time reduction corresponds to approximately 50% of the required time in case the same server would be used for captured data as for the emulated sound sources.

### 6.3. Defining a Microphone Array

The UMAP and the Quarter array show comparable results for both the emulated sound sources and the captured acoustic data. A set of metrics have been defined in Section 3 describing the ability of an implemented algorithm to find the AoA of a sound source on a given microphone array. Although a single metric describing the performance of microphone array would be ideal, the set of metrics allow to define the boundary conditions of the beamwidth, the PSLL, the directivity and the FI to the given application of use. The effects of the boundaries on finding a sound source is listed below.

- The beamwidth: a thinner beamwidth allows to find a sound source in a smaller angular region. However, a thinner beamwidth also requires more microphones and thus also more processing capabilities.
- The Directivity: A higher directivity allows to predict with a higher probability the AoA of a sound source for a given microphone array.
- The PSLL: in order to be able to find a sound source in a given AoA, a negative PSLL must be obtained. More microphones and processing capabilities are required to obtain lower and thus more optimal PSLL values.
- The FI: this metric is related to the beamwidth and the PSLL. A positive value of the FI is to be obtained so that a sound source can be found, where a value of 1 is preferred at the expense of more microphones and processing capabilities.

The metrics can be used in case that a sound source is to be found in only one given AoA. The steered metrics allow to define the angular range in which the microphone array is expected to operate. The variation of the Q3 factor and the minimum and maximum values of a given metric ideally have to be zero in case the probability of finding a sound source in all desired AoA is expected to be equal. The latter combined with optimal boundaries have to be weighed up against the number of microphones, the desired beamforming algorithm and the placement of the microphones. It is also possible to weigh up the metrics against each other with a given number of microphones. More important metric can be optimized at the expense of other metrics while keeping the same number of microphones and beamforming algorithm.

## 7. Conclusions

In this paper the CABE platform which allows to assess the performance of a user defined microphone array design along with the selected signal processing chain is proposed. The emulator is built on the D&S beamforming principle where the order of signal processing operations can be changed. The effects of non-idealities of the microphones and the effects of several signal processing chains affect the results and thus the probability of finding a sound source. The effects of fixed point computation compared to results obtained with double precision arithmetic are also outlined. The platform is capable of taking advantage of multicore environments and it is also possible to deploy the back-end beamforming emulator on multiple machines so that concurrent emulation requests can be computed simultaneously. The compute-time and the machine properties on which the computations are performed are logged and shown to the user via the CABE client application. The different far-field steering methods enable to compute beamforming algorithms in both 2D and 3D. The CABE client application allows to issue new requests and to visualize the results using several display modes. An HDL package generator is also included so that development towards FPGA implementations is facilitated. More importantly, the emulator platform also allows to process samples captured from a microphone array and to generate the appropriate results. With this last possibility one can compare results from emulated sound sources with the results from captured samples. At last, assessing the performance of a given microphone array with the chosen signal processing techniques is facilitated with the use of the metrics. These metrics depict the effectiveness of locating an acoustic sound source with a given frequency. The steered metrics allow to assess the performance of an array in all the angles of interest, where the minimum and maximum computed steered response power results along with the average and 75 percentiles are computed. The beamforming emulator is currently used in our Reconfigurable Architectures, Parallel Processing & Telecommunications Oriented Research (RAPPTOR) team and additional possibilities are added on a regular basis. The platform can be accessed at https://projects.rapptor.vub.ac.be/CABE/. The current platform allows to emulate microphone arrays with acoustic waves of different frequencies in an open field without reverberations. Adding acoustic reverberation techniques would help to mimic a real environment and to evaluate the response of given microphone array. Obtaining some results requires a computation time of several hours. This could lead to a long waiting time in case a lot of emulations are requested. Adding support for Graphics Processing Unit (GPU) and or FPGA support in the emulator would drastically reduce the compute-time. Updates will be added to this website, where the client and the visualization tools can be downloaded as well.

## Figures and Tables

**Table 1:** Machine on which the emulations are performed. The first machine is the only machine completely dedicated for the emulations while the three other virtual machines are running in a VMWare ESXi environment.

Machine Type	Number of Cores	CPU Model	CPU Frequency (GHz)	RAM (GB)
Virtual server 1	8 (16 hyper threaded)	Intel Xeon^®^E5530 CPU	2.4	12
Virtual server 2	8	Intel Xeon^®^Gold 5118 CPU	2.3	16
Virtual server 3	8	Intel Xeon^®^Gold 5118 CPU	2.3	16
Virtual server 4	4	Intel Xeon^®^Gold 5118 CPU	2.3	16

**Table 2:** List of operations with the corresponding time required to complete each operation. The machine on which the operation has been computed is listed in the second column. The abbreviations “aessp” and “aesmp” respectively denote “acoustic emitting source single position” and “acoustic emitting source multiple positions”.

Emulation Configuration	Machine	Completion Time (s)
UMAP subarray 1, aessp	Virtual server 2/3	309
UMAP subarray 2, aessp	Virtual server 1	602
UMAP all subarrays, aessp	Virtual server 2/3	487
Quarter subarray 1, aessp	Virtual server 2/3	396
Quarter subarray 2, aessp	Virtual server 1	558
Quarter all subarrays, aessp	Virtual server 2/3	549
UMAP subarray 1, aesmp	Virtual server 1	25,089
UMAP subarray 2, aesmp	Virtual server 4	22,241
UMAP all subarrays, aesmp	Virtual server 2/3	19,152
Quarter subarray 1, aesmp ($360^{\circ }$)	Virtual server 2/3	17,803
Quarter subarray 2, aesmp ($360^{\circ }$)	Virtual server 4	29,659
Quarter all subarrays, aesmp ($360^{\circ }$)	Virtual server 2/3	24,838
Quarter subarray 1, aesmp ($90^{\circ }$)	Virtual server 2/3	13,744
Quarter subarray 2, aesmp ($90^{\circ }$)	Virtual server 2/3	14,103
Quarter all subarrays, aesmp ($90^{\circ }$)	Virtual server 1	28,638

**Table 3:** List of operations with the corresponding time required to complete each operation. The machine on which the operation has been computed is listed between brackets next to the compute-time.

Emulation Configuration	Completion Time Capturing (s)	Completion Time Simulation (s)
UMAP 17.1 kHz	4 (Virtual server 2/3)	11 (Virtual server 1)
UMAP 25.1 kHz	6 (Virtual server 1)	8 (Virtual server 2/3)
UMAP 27 kHz	4 (Virtual server 2/3)	8 (Virtual server 4)
Quarter 20 kHz	4 (Virtual server 2/3)	15 (Virtual server 1)
Quarter 23.7 kHz	4 (Virtual server 4)	12 (Virtual server 2/3)
Quarter 36.2 kHz	5 (Virtual server 2/3)	12 (Virtual server 4)
